# SUMO enhances unfolding of SUMO–polyubiquitin-modified substrates by the Ufd1/Npl4/Cdc48 complex

**DOI:** 10.1073/pnas.2213703120

**Published:** 2022-12-27

**Authors:** Hyein G. Lee, Abigail A. Lemmon, Christopher D. Lima

**Affiliations:** ^a^Biochemistry, Structural Biology, Cell Biology, Developmental Biology and Molecular Biology (BCMB) Allied Program, Weill Graduate School of Medical Sciences, Cornell University, New York, NY 10065; ^b^Structural Biology Program, Sloan Kettering Institute, New York, NY 10065; ^c^Tri-Institutional PhD Program in Chemical Biology, Memorial Sloan Kettering Cancer Center, New York NY 10065; ^d^HHMI, New York, NY 10065

**Keywords:** quality control, ubiquitin, segregase, SUMO, stress

## Abstract

The Ufd1/Npl4/Cdc48 complex is a universal protein segregase that plays key roles in eukaryotic cellular processes. Its functions orchestrating the clearance or removal of polyubiquitylated targets are established; however, prior studies suggest that the complex also targets substrates modified by the ubiquitin-like protein SUMO. Here, we show that interactions between Ufd1 and SUMO enhance unfolding of substrates modified by SUMO–polyubiquitin hybrid chains by the budding yeast Ufd1/Npl4/Cdc48 complex compared to substrates modified by polyubiquitin chains, a difference that is accentuated when the complex has a choice between these substrates. Incubating Ufd1/Npl4/Cdc48 with a substrate modified by a SUMO–polyubiquitin hybrid chain produced a series of single-particle cryo-EM structures that reveal features of interactions between Ufd1/Npl4/Cdc48 and ubiquitin prior to and during unfolding of ubiquitin. These results are consistent with cellular functions for SUMO and ubiquitin modifications and support a physical model wherein Ufd1/Npl4/Cdc48, SUMO, and ubiquitin conjugation pathways converge to promote clearance of proteins modified with SUMO and polyubiquitin.

## Significance

Protein quality control pathways enhance protein activities by promoting maturation, preventing aggregation, or by targeting proteins or protein complexes for degradation by pathways such as the ubiquitin–proteasome pathway. Ubiquitin and ubiquitin-like protein SUMO conjugation pathways cooperate to ensure quality control after cellular stresses including heat shock or DNA damage to sometimes generate SUMO–polyubiquitin hybrid substrates. Defects in these pathways contribute to disease. Our results show that these substrates are preferentially targeted by Ufd1/Npl4/Cdc48, a protein segregase complex known to target polyubiquitin-conjugated proteins or subunits to remove them from complexes, to extract them from membranes, or to unfold them. Our study illustrates potential regulation in protein quality control and a biochemical basis for convergence of SUMO and ubiquitin conjugation pathways.

Ubiquitin and small ubiquitin-like modifier (SUMO) conjugation represent two essential post-translational modifications that participate in nearly every cellular process (1–4). Substrate conjugation by ubiquitin and ubiquitin-like proteins such as SUMO requires the sequential activities of three-enzyme cascades involving E1 activating enzymes, E2 conjugating enzymes, and E3 protein ligases that result in covalent modification of targets, principally on substrate lysine residues. These priming conjugation events can be further remodeled by ligases and proteases to generate ubiquitin and ubiquitin-like polymeric chains with different topologies (4), each of which has the potential to signal through factors that recognize different chain topologies. Several studies suggest overlap in ubiquitin and SUMO conjugation pathways in various cellular processes including heat shock and DNA damage responses and the maintenance of subcellular structures including promyelocytic leukemia or PML bodies (5–11).

SUMO-targeted ubiquitin ligases (STUbLs) represent a conserved class of E3 ubiquitin ligases that target poly-SUMO-conjugated proteins for modification by ubiquitin (12, 13). While STUbLs can target proteins in the absence of SUMO (14), specificity for SUMO chains is achieved by E3 ligase subunits that contain tandem SUMO Interaction Motifs (SIMs) (9, 15). Key phenotypes of STUbL dysfunction include genomic instability, hypersensitivity to genotoxins, and accumulation of high molecular weight SUMO chains (16, 17). Several lines of evidence suggest that STUbLs provide a means to clear SUMO-conjugated proteins after events such as heat shock or DNA damage (18–20). Biochemically, STUbL-mediated ubiquitin conjugation of SUMO-modified targets can result in dual modification with SUMO and ubiquitin on different lysines in the target complex, or hybrid chain modification with polyubiquitin conjugated to a SUMO chain. Proteomics studies revealed the presence of ubiquitin-conjugated SUMO in vivo, lending support to the idea that hybrid chains could serve as intermediates in protein clearance (21, 22).

Potential readers of substrates modified with SUMO–polyubiquitin hybrid chains include RAP80 in the mammalian DNA damage response (23) and the Ufd1/Npl4/Cdc48 complex in budding and fission yeast (24, 25). The Ufd1/Npl4/Cdc48 complex is a crucial component of ubiquitin-mediated protein metabolism as the universal segregase that removes targets marked with polyubiquitin from complexes and membranes. It is composed of the Ufd1/Npl4 dimer adaptor, which determines substrate specificity, and the Cdc48 hexamer, a AAA+ protein that couples adenosine triphosphate (ATP) hydrolysis to protein unfolding (26–28). Recent structural studies have demonstrated that lysine 48-linked polyubiquitin acts as a recruitment signal and the initiation site for unfolding by the complex, as a peptide corresponding to unfolded ubiquitin was observed threaded across the Npl4 surface and into the channel formed by the Cdc48 hexamer (28). Subsequent studies revealed a complex interplay between interactions with polyubiquitylated substrates and productive unfolding, namely that Ufd1/Npl4/Cdc48 can unfold any ubiquitin within the K48-linked chain, but productive substrate unfolding only occurs after unfolding a ubiquitin molecule that is proximal and N-terminal but not C-terminal to the substrate (29). These studies suggest that the topology of substrate and polyubiquitin defines whether a substrate can be unfolded and that the search for substrate proximal ubiquitin may be a rate-limiting step.

Ufd1 in budding yeast possesses a C-terminal SIM that was uncovered in the anti-recombinogenic helicase Srs2, where the SIM plays a critical role in the recognition of SUMO-modified proliferating cell nuclear antigen (30, 31). The analogous C-terminal SIM in fission yeast Ufd1 interacts with SUMO and contributes to maintenance of genome integrity (24). Colocalization of Ufd1 to SUMO foci increases during the DNA damage response, and the resolution of SUMO foci appears dependent on the C-terminal SIM of Ufd1 as its deletion results in an increase in intensity of DNA damage-associated SUMO foci in fission yeast (32). Furthermore, deletion of the SIM is genetically epistatic with STUbL mutants with respect to the DNA damage response (32). Consistent with these observations, substantial overlap exists in perturbations of the proteome that result from deletion of the Ufd1 C-terminus or disruption of STUbL function, leading to the hypothesis that Ufd1 and STUbLs work in the same pathways (33).

Here, we set out to reconstitute substrates modified with SUMO and polyubiquitin to mimic SUMO-modified proteins after STUbL-mediated ubiquitylation. We then used these substrates to determine their propensities for unfolding by the *Saccharomyces cerevisiae* Ufd1/Npl4/Cdc48 complex to define contributions of SUMO to this process. We show that compared to the canonical polyubiquitin-only-modified substrates, the Ufd1/Npl4/Cdc48 complex preferentially unfolds SUMO–polyubiquitin dual-modified substrates in a Ufd1 SIM and SUMO-dependent manner. This SUMO-dependent unfolding activity is also conserved in the *Schizosaccharomyces pombe* Ufd1/Npl4/Cdc48 complex. Additionally, we present previously unreported single-particle cryogenic electron microscopy (cryo-EM) structures of the Ufd1/Npl4/Cdc48 complex with a SUMO–polyubiquitin substrate in multiple states showing the complex prior to and during ubiquitin unfolding. Our results support a model in which SUMO enhances unfolding by increasing interactions with the substrate, potentially facilitating the search for substrate proximal ubiquitin.

## Results

### In Vitro Synthesis and Characterization of SUMO–Polyubiquitin Dual-Modified Substrates.

**Fig. 1.**
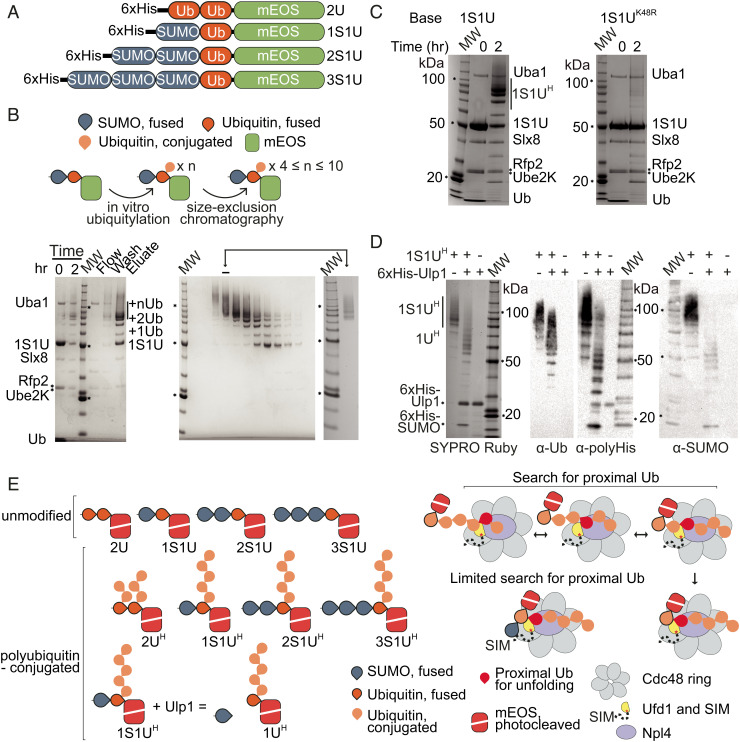
Synthesis and characterization of SUMO/polyubiquitin dual-modified substrates. (*A*) Schematics of substrates. Two ubiquitin molecules (2U), one SUMO and one ubiquitin (1S1U), two SUMO and one ubiquitin (2S1U), or three SUMO and one ubiquitin (3S1U) were fused end to end with the N-terminus of mEOS (*B*) *Top*: schematic of ubiquitylation and purification of substrates. *Bottom Left*: Substrates were ubiquitylated with E1 Uba1, E2 Ube2K, and STUbL E3 ubiquitin ligase Slx8-Rfp2. Ubiquitylation of 1S1U shown with ubiquitylated species (+nUb) labeled. Molecular weight marker (MW) is Invitrogen BenchMark Protein Ladder, markers for 100, 50, and 20 kDa are noted with text and/or asterisk (*) in panels *B–D*. *Bottom Right*: Substrates were purified by size exclusion chromatography to select for substrates with chains estimated to include 4 to 10 ubiquitin molecules. *Inset*: substrate pool after purification. (*C*) Lysine 48 of the substrate ubiquitin is required for efficient chain elongation. *Left*: Ubiquitylation of 1S1U substrate as in *B*. *Right*: Substrate containing a K48R mutation was subjected to ubiquitin conjugation. (*D*) SUMO is not the primary site for ubiquitin conjugation. 1S1U^H^ substrate was digested with Ulp1 to liberate SUMO, generating 1U^H^. SYPRO Ruby staining and western blotting for the polyhistidine tag (α-polyHis), SUMO (α-SUMO), and ubiquitin (α-Ub) in the presence or absence of Ulp1 and substrate. (*E*) *Left*: Composition of substrates is denoted by S for SUMO or U for ubiquitin and numbering of fused molecules. Superscript H denotes substrates containing ubiquitin chains estimated to be 4 to 10 ubiquitin molecules long. *Right*: Model of search for proximal ubiquitin and the proposed limitations on the search for proximal ubiquitin when SUMO is present in substrate.

We generated polyubiquitin-modified and SUMO–polyubiquitin dual-modified substrates by modifying a protocol for creating polyubiquitylated Ufd1/Npl4/Cdc48 substrates using tandem ubiquitin molecules fused end to end with the N-terminus of monomeric Eos Fluorescent protein (mEOS) (27). Our protocol employs a similar strategy, but now includes additional fusions of Smt3 (yeast SUMO referred to hereafter as SUMO). We generated linear fusions of either two ubiquitin moieties or fusions of one, two, or three SUMO moieties followed by one ubiquitin moiety ahead of the unfoldase assay reporter, mEOS (Fig. 1*A*). These proteins were subsequently used to generate substrates with extended Lys48-linked polyubiquitin chains by incubation with a Lys48-specific chain-elongating E2 Ube2K that contains an acceptor face specific for conjugating ubiquitin to ubiquitin (34) and the STUbL E3 ubiquitin ligase Slx8-Rfp2 (13) to promote chain elongation.

The ubiquitin conjugation reaction produced a distribution of polyubiquitin chain lengths (Fig. 1*B*). After purifying the modified substrate from components of the conjugation reaction through Ni-nitriloacetic acid (NTA) affinity with the N-terminal polyhistidine tag, the substrate population was separated by ubiquitin chain lengths using size-exclusion chromatography. Substrates with ubiquitin chains estimated to include 4 to 10 ubiquitin molecules were pooled and used for subsequent assays, unless otherwise noted (Fig. 1*B*).

Consistent with ubiquitin specificity and chain elongation activities of Ube2K, a substrate containing a single Lys48Arg mutation within ubiquitin was deficient in chain elongation in these assays (Fig. 1*C*). To confirm a preference for adding ubiquitin chains to ubiquitin and not to the N-terminal SUMO, substrates were incubated with SUMO protease Ulp1 to cleave off the N-terminal SUMO. This showed that SUMO can be modified with ubiquitin after incubation with Ube2K (Fig. 1*D*), but that the majority of polyubiquitin species remain at higher molecular weight (above 50 kDa) consistent with the majority of ubiquitin chains emanating from the ubiquitin molecule in substrates containing fusions between SUMO, ubiquitin, and mEOS. The *Left* panel of Fig. 1*E* presents a summary depicting the substrates generated for this study, where the name includes S or U to denote SUMO or ubiquitin, a number to denote the number of molecules in the N-terminal fusion, and a superscript H to denote substrates modified by ubiquitin chains estimated at 4 to 10 molecules in length.

### SUMO and the Ufd1 SIM Enhance Substrate Unfolding by Ufd1/Npl4/Cdc48.

The Ufd1/Npl4/Cdc48 complex is a protein unfoldase (27, 28, 35). To determine if substrate unfolding by Ufd1/Npl4/Cdc48 was altered by the inclusion of SUMO, polyubiquitin, or SUMO–polyubiquitin on the substrate, we generated substrates fused to mEOS, an unfoldase reporter (27). This unfoldase reporter is a photocleaved fluorescent protein that once unfolded cannot refold. The loss of fluorescence is therefore interpreted as unfolding. The rate of unfolding observed in this assay can reflect any number of rate-limiting steps such as substrate association, substrate dissociation, as well as the likelihood of substrate engagement and unfolding following association.

**Fig. 2.**
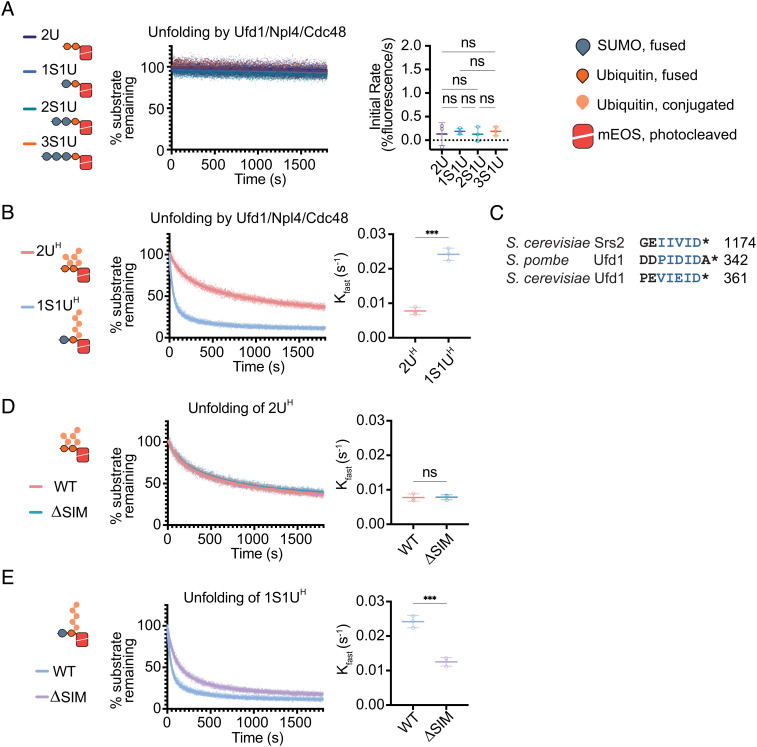
Unfolding depends on polyubiquitin, SUMO, and SIM. (*A*) Non-polyubiquitylated substrates are not readily unfolded. Loss of fluorescence was measured for non-ubiquitylated substrates 2U, 1S1U, 2S1U, and 3S1U by WT Ufd1/Npl4/Cdc48. (*B*) Unfolding increases in the presence of SUMO. Unfolding of either 2U^H^ or 1S1U^H^ by WT Ufd1/Npl4/Cdc48. (*C*) Alignment of C-terminal residues of Srs2 from *S. cerevisiae*, and Ufd1 from *S. pombe* and *S. cerevisiae*. Blue lettering indicates SIM. Asterisk (*) denotes the C-terminus of the protein. Unfolding of 2U^H^ (*D*) or 1S1U^H^ (*E*) by WT Ufd1/Npl4/Cdc48 (WT) or Ufd1^ΔSIM^/Npl4/Cdc48 where the SIM was deleted from Ufd1 (ΔSIM). Values were normalized to background fluorescence in the absence of ATP. Plot of three replicates with fit of linear regression (*A*) or two-phase nonlinear regression (*B*, *D*, and *E*). Initial rate determined using the first 30 s of linear fit or K_fast_ (s^−1^) determined using two-phase exponential decay fit. Error bars represent SD. *P* values calculated by one-way ANOVA with Tukey’s test (*A*) or unpaired two-tailed *t* test (*B*, *D*, and *E*); *< 0.05, **P < 0.01, ***P < 0.001, ns (not significant).

We started by determining if the Ufd1/Npl4/Cdc48 complex could unfold mEOS substrates containing SUMO–Ub or tandem Ub lacking polyubiquitin chains. Unfolding was not observed for any substrate lacking a polyubiquitin chain (Fig. 2*A*). While Ufd1/Npl4/Cdc48 can bind substrates containing SUMO without polyubiquitylation (*SI Appendix*, Fig. S1*A* and Table S1), unfolding was not observed suggesting that binding of SUMO is not sufficient to promote unfolding. These results are consistent with prior work showing that ubiquitin chains of at least four molecules are required for unfolding (28, 29, 36).

As observed previously, the Ufd1/Npl4/Cdc48 complex readily unfolds substrates containing polyubiquitin chains, as indicated by the loss of fluorescence observed for 2U^H^ and 1S1U^H^ substrates (Fig. 2*B*). Notably, the substrate containing both SUMO and polyubiquitin is unfolded more proficiently than the canonical polyubiquitin-only substrate. To determine if increased rates observed for unfolding of 1S1U^H^ are due to SUMO, we reconstituted a Ufd1/Npl4/Cdc48 complex that contained a five-amino acid deletion in Ufd1 to remove the SIM that is analogous to C-terminal SIMs in Srs2 and fission yeast Ufd1 (Fig. 2*C*). The mutant Ufd1^Δ^^SIM^/Npl4/Cdc48 complex exhibits similar rates of unfolding for the 2U^H^ polyubiquitin-only substrate compared to wild-type (WT) (Fig. 2*D*). In contrast, unfolding rates for the 1S1U^H^ SUMO–polyubiquitin substrate are slower for Ufd1^ΔSIM^/Npl4/Cdc48 compared to WT (Fig. 2*E*), consistent with the increase in high molecular weight SUMO conjugates observed in vivo with Ufd1^Δ^^SIM^ (33).

**Fig. 3.**
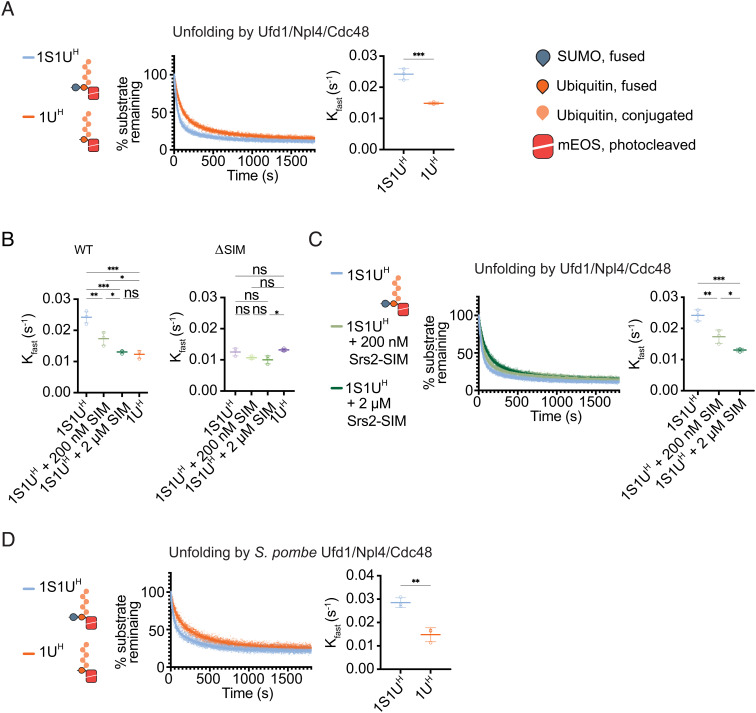
Rate of Ufd1/Npl4/Cdc48 unfolding is enhanced in a SUMO- and SIM-dependent manner. (*A*) Unfolding of 1S1U^H^ or 1U^H^ (Ulp1-treated 1S1U^H^) by WT Ufd1/Npl4/Cdc48. (*B*) Comparison of K_fast_ values for WT or ΔSIM complexes for 1S1U^H^, 1S1U^H^ + 200 nM SIM peptide, 1S1U^H^ + 2 µM SIM peptide, or 1U^H^. (*C*) Unfolding of 1S1U^H^ by WT Ufd1/Npl4/Cdc48 in presence of a competing SIM peptide. 200 nM or 2 µM of SIM peptide derived from the C-terminus of Srs2 (residues 1,107 to 1,174) was added to the unfolding reaction of 1S1U^H^. (*D*) Unfolding by *S. pombe* WT Ufd1/Npl4/Cdc48 of 1S1U^H^ or 1U^H^. Values were normalized to background fluorescence in the absence of ATP. Plot of three replicates with fit of two-phase nonlinear regression. K_fast_ (s^−1^) determined using two-phase exponential decay fit. Error bars represent SD. *P* values calculated by unpaired two-tailed *t* test (*A* and *D*) or one-way ANOVA with Tukey’s test (*B* and *C*); *P < 0.05, **P < 0.01, ***P < 0.001, ns (not significant).

If the C-terminal SIM recognizing SUMO is responsible for enhanced unfolding, we reasoned rates should decrease if SUMO was removed from the 1S1U^H^ substrate. Indeed, removal of SUMO by pre-treatment with SUMO protease Ulp1 decreased the rate of unfolding by Ufd1/Npl4/Cdc48 (Fig. 3*A*) to levels observed for Ufd1^Δ^^SIM^/Npl4/Cdc48 with the 1S1U^H^ substrate (Fig. 3 *A* and *B*). To further confirm that rate enhancement is mediated by SIM/SUMO interactions, reactions were conducted in the presence of a peptide derived from the C-terminal region of Srs2 that binds to SUMO via a C-terminal SIM similar to that of Ufd1 (30, 31). As predicted, rates decreased in a SIM peptide concentration-dependent manner, with 2 µM SIM peptide reducing the rate to that observed for 1S1U^H^ after removal of SUMO by the Ulp1 SUMO protease (Fig. 3 *B* and *C*). In summary, unfolding was reduced to similar rates by removing SUMO from the 1S1U^H^ substrate, by deletion of the Ufd1 SIM, or by addition of a competing SIM (Fig. 3*B*). Importantly, slowing rates by adding SIM peptide or removing SUMO were specific to Ufd1 SIM as they did not alter rates of unfolding by Ufd1^Δ^^SIM^/Npl4/Cdc48 (Fig. 3*B*). This effect appears conserved between budding and fission yeast systems as the *S. pombe* Ufd1/Npl4/Cdc48 complex also exhibits a decreased rate of unfolding of the Ulp1-treated 1S1U^H^ substrate (1U^H^) relative to the untreated 1S1U^H^ substrate (Fig. 3*D*).

To determine the contribution of binding to unfolding, biolayer interferometry was employed. Various available models were used to analyze data, but a 2:1 heterogeneous ligand fit appeared to fit the data best, perhaps consistent with its ability to deal with the complexity of the analyte (SUMO/ubiquitin/polyubiquitin and their combinations as epitopes) and the number of potential binding sites in the ligand (Ufd1 SIM and Ufd1/Npl4 ubiquitin interaction sites). Consistent with interactions being dependent on the Ufd1 SIM and substrate SUMO, improved binding (K_D_) and faster association rates were observed for Ufd1/Npl4 and 1S1U^H^ relative to 2U^H^ or 1U^H^ (1S1U^H^ after removing SUMO with Ulp1) but not for Ufd1^Δ^^SIM^/Npl4 suggesting that the SIM was important for this effect (*SI Appendix*, Fig. S1 *B*–*D* and Table S1). This result is consistent with SIM-dependent interactions with the 1S1U substrate in the absence of a polyubiquitin chain where deletion of the SIM led to a 100-fold binding defect (*SI Appendix*, Fig. S1*A* and Table S1). We were unable to achieve satisfactory fits for substrates lacking polyubiquitin but containing more than one SUMO, but unfolding rates were not altered if additional molecules of SUMO are present in the context of 2S1U^H^ and 3S1U^H^ (*SI Appendix*, Fig. S2), consistent with polyubiquitin-dependent unfolding and polyubiquitin contributing to binding more than SUMO (*SI Appendix*, Fig. S1 and Table S1).

### Ufd1/Npl4/Cdc48 Preferentially Unfolds SUMO–Polyubiquitin-Modified Substrates in a Mixed Substrate Pool.

Results thus far suggest that Ufd1/Npl4/Cdc48 may prefer substrates containing SUMO–polyubiquitin hybrid chains over those containing polyubiquitin chains. To assess this prediction more directly, we next measured unfolding rates in mixed substrate pools containing 1U^H^ and 1S1U^H^ substrates as either native (green mEOS—denoted as ^G^) or photocleaved substrate (red mEOS—denoted as ^R^) to selectively monitor the unfolding of photocleaved substrate in the presence of native mEOS substrate. While native mEOS may refold after unfolding, photocleaved mEOS cannot.

**Fig. 4.**
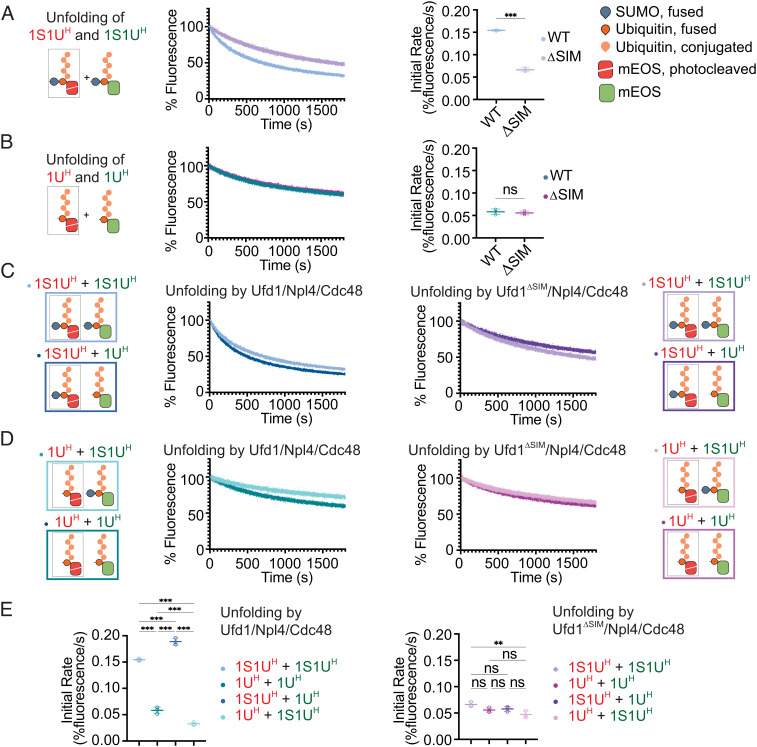
Ufd1/Npl4/Cdc48 preferentially unfolds SUMO–polyubiquitin substrates in a mixed substrate pool. Pooled substrates contained activated mEOS (denoted by red cartoon and text) or native mEOS (denoted by green cartoon and text). Unfolding of (*A*) 1S1U^H^-mEOS^R^ and 1S1U^H^-mEOS^G^ or (*B*) 1U^H^-mEOS^R^ and 1U^H^-mEOS^G^ by WT and ΔSIM Ufd1/Npl4/Cdc48. (*C*) Unfolding of 1S1U^H^-mEOS^R^ and 1S1U^H^-mEOS^G^ or 1U^H^-mEOS^G^ by WT (*Left*) and ΔSIM (*Right*) Ufd1/Npl4/Cdc48. (*D*) Unfolding of 1U^H^-mEOS^R^ and 1S1U^H^-mEOS^G^ or 1U^H^-mEOS^G^ by WT (*Left*) and ΔSIM (*Right*) Ufd1/Npl4/Cdc48. Values were normalized to background fluorescence in the absence of ATP. Plot of three replicates. (*E*) Initial rates of unfolding for WT (*Left*) and ΔSIM mutant (*Right*) Ufd1/Npl4/Cdc48 in *A*–*D*. Initial rate determined using linear fit of the first 30 s of unfolding. Error bars represent SD. *P* values calculated by unpaired two-tailed *t* test (*A* and *B*) or one-way ANOVA with Tukey’s test (*E*); *P < 0.05, **P < 0.01, ***P < 0.001, ns (not significant).

To ensure changes in rate reflect preferences for the polyubiquitin-only 1U^H^ or SUMO–polyubiquitin hybrid 1S1U^H^ substrates, reactions were conducted with both substrates in excess and equimolar under multiple-turnover conditions relative to Ufd1/Npl4/Cdc48. Consistent with prior results, unfolding photocleaved 1S1U^H^-mEOS^R^ in the presence of 1S1U^H^-mEOS^G^ substrate remained SIM-dependent as evidenced by an increased rate for Ufd1/Npl4/Cdc48 compared to Ufd1^Δ^^SIM^/Npl4/Cdc48 (Fig. 4*A*) while unfolding rates for these two complexes with 1U^H^-mEOS^R^ in presence of 1U^H^-mEOS^G^ were comparable (Fig. 4*B*). Under mixed substrate conditions, the rate of unfolding by Ufd1/Npl4/Cdc48 of 1S1U^H^-mEOS^R^ increased in the presence of 1U^H^-mEOS^G^ while the rate of unfolding of 1U^H^-mEOS^R^ decreased in the presence of 1S1U^H^-mEOS^G^ (Fig. 4 *C–E*), effects that diminished in the absence of the SIM in Ufd1^Δ^^SIM^/Npl4/Cdc48 (Fig. 4 *C–E*). Together, these data suggest that Ufd1/Npl4/Cdc48 not only unfolds the SUMO–polyubiquitin 1S1U^H^ substrate more readily than the polyubiquitin-only 1U^H^ substrate, but that it exhibits a preference for the SUMO–polyubiquitin substrate when challenged with a mixture of substrates containing hybrid and homotypic chains, a preference that is SIM- and SUMO-dependent.

### Structures of Ufd1/Npl4/Cdc48 in Complex with SUMO–Polyubiquitin-Modified Substrate 1S1U^H^-mEOS.

Previous structures of Ufd1/Npl4 and Ufd1/Npl4/Cdc48 were determined in the presence of a polyubiquitin substrate (28, 37). Any ubiquitin in the chain can be sampled for unfolding; however, productive unfolding of the substrate is more likely to occur after unfolding the proximal ubiquitin within the Lys48-linked ubiquitin chain (28, 29). Given the biochemical preference for SUMO–polyubiquitin substrates and putative location of the Ufd1 SIM in the complex, we next sought to determine if contacts to Ufd1 might enhance or alter interactions between Ufd1/Npl4/Cdc48 and a SUMO–polyubiquitin substrate (Fig. 1*E*).

**Fig. 5.**
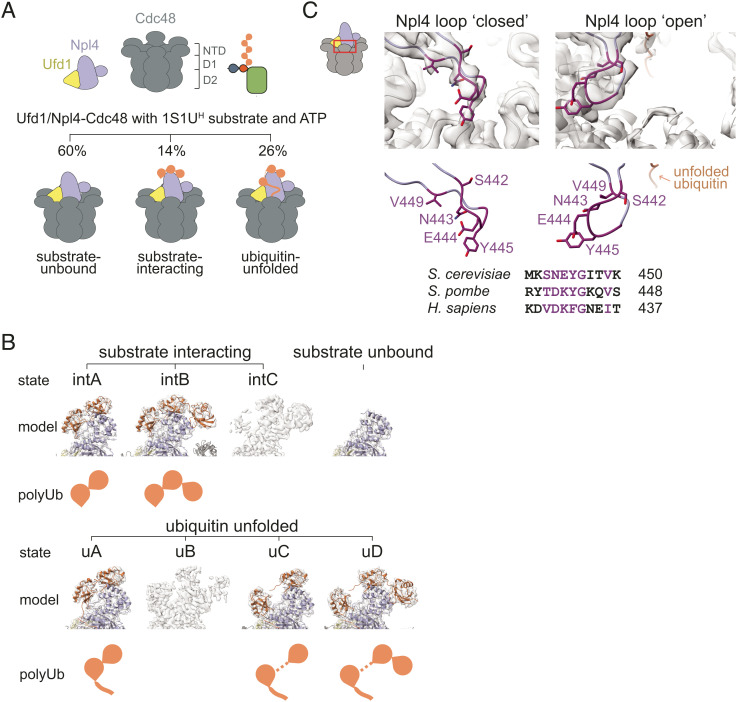
Cryo-EM analysis of Ufd1/Npl4/Cdc48 in process of unfolding a SUMO–polyubiquitin substrate. (*A*) *Top*: cartoon of components in sample. 1S1U^H^ was generated as in Fig. 1 and added prior to photocleavage to allow multiple rounds of unfolding. Components were preincubated with ATP prior to vitrification. *Bottom*: cartoon showing the three main classes of particles observed as described by their relation to the substrate—substrate-unbound, substrate-interacting, and ubiquitin-unfolded. (*B*) EM density and models of sub-states in substrate-interacting (denoted with the prefix “int”) and ubiquitin-unfolded (denoted with the prefix “u”) classes. Subclassification revealed at least eight states within the sample. Classes differ in the net number of folded ubiquitin molecules, their position atop Ufd1/Npl4, and the presence or absence of unfolded ubiquitin—these differences are represented by the “polyUb” cartoon. In five states that could be modeled, a cartoon representation of the model (colored; Npl4 in light blue, ubiquitin in orange, and Ufd1 in yellow) is shown overlayed with EM density (in grey). (*C*) EM density and model of the Npl4 loop (*Top*) and sequence alignment of the Npl4 loop (*Bottom*) with conserved residues in dark purple. Conserved Npl4 loop adopts discrete positions: closed, occluding the central pore of the Cdc48 hexamer (as seen in substrate-unbound and substrate-interacting classes) or open, allowing access to the central pore (as seen in ubiquitin-unfolded class).

**Fig. 6.**
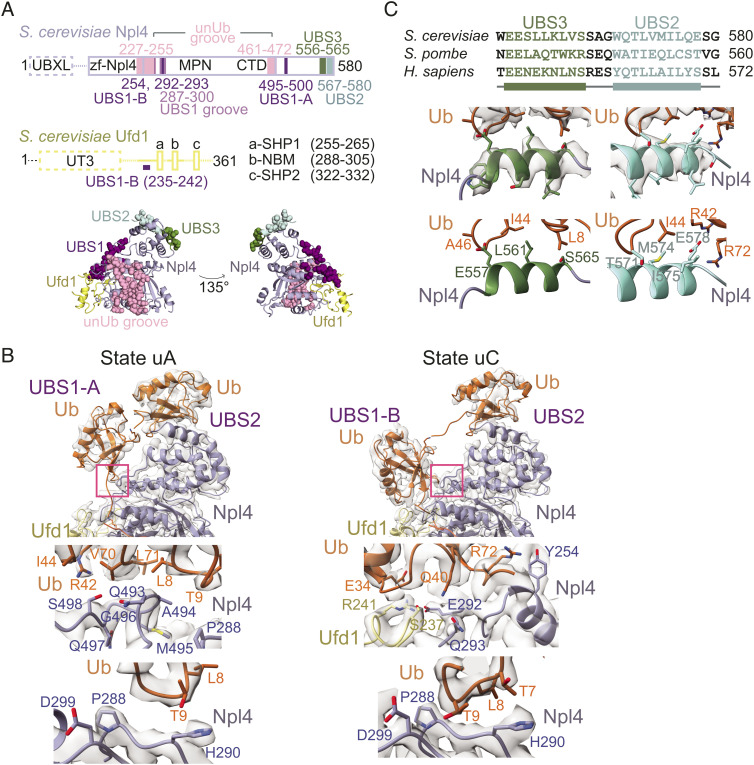
Sites of ubiquitin interaction in Ufd1/Npl4/Cdc48. (*A*) Ufd1/Npl4 domain organization. Solid lines indicate regions modeled in cryo-EM reconstructions. The four sites of ubiquitin association are colored and residues of the sites are shown as spheres in the cartoon representation below. Ufd1 is in yellow and Npl4 is in light blue. (*B*) *Top*: Close-up of ubiquitin in UBS1 in EM density maps and models of states uA and uC. uA represents states where ubiquitin occupies UBS1-A (also observed in states intA and intB) while uC represents states where ubiquitin occupies UBS1-B (also observed in uD). Pink boxes highlight sites of ubiquitin interaction shown in *Lower* panels. *Middle Left*: Interactions between ubiquitin in UBS1-A and Npl4. Side chains of residues in ubiquitin and Npl4 that are positioned to mediate the association are shown and labeled. *Middle Right*: Interactions between ubiquitin in UBS1-B and Ufd1/Npl4. Side chains of residues in ubiquitin, Npl4 and Ufd1 that mediate contacts are shown and labeled. *Bottom*: The Npl4 pivot groove. Positioning of the β1–β2 loop of ubiquitin at the Npl4 pivot groove in UBS1-A (*Left*) and UBS1-B (*Right*) are shown. (*C*) EM density map and models representing UBS3 and UBS2 as derived from state uD, and their sequence alignment. UBS3 and UBS2 include two helices separated by a short linker that interact with tandem ubiquitin molecules in the Lys48 chain.

*S. cerevisiae* Ufd1/Npl4/Cdc48 was prepared and incubated in the presence of 1S1U^H^-mEOS and ATP·Mg and analyzed by single-particle cryo-electron microscopy (*SI Appendix*, Fig. S3 and Table S2). More than 700k particles from three datasets were combined and segregated by 3D classification into three major classes of the Ufd1/Npl4/Cdc48 complex: no ubiquitin nor substrate-bound (“substrate-unbound”), bound to substrate prior to unfolding (“substrate-interacting”), or bound to substrate and unfolding a ubiquitin (“ubiquitin-unfolded”) (Fig. 5*A* and *SI Appendix*, Figs. S4 and S5). Further rounds of classification and focused refinement of substrate-unbound, substrate-interacting and ubiquitin-unfolded particles produced a substrate-unbound structure with a resolution range of 2.8 to 7.6 Å, three substrate-interacting states (states intA, intB, and intC)—two of which were used to build atomic models (intA and intB at overall resolution ranges of 3.2 to 8.2 Å and 3.2 to 10.5 Å, respectively)—and four ubiquitin-unfolded states (states uA, uB, uC, and uD)—three of which were used to build atomic models (uA, uC, and uD at resolution ranges of 3.3 to 9.4 Å, 3.3 to 8.7 Å, and 3.5 to 8.7 Å, respectively) (Fig. 5*B* and *SI Appendix*, Figs. S4 and S5). In addition to three states between Ufd1/Npl4/Cdc48 and substrates not previously reported, these structures reveal a ubiquitin interaction site that coordinates distal ubiquitin in the K48-linked chain and atomic features for Ufd1 not modeled in prior structures that reveal contacts between Ufd1 and ubiquitin that appear to contribute to ubiquitin destabilization. Up to five distinct ubiquitin binding sites (UBSs) are now resolved on Ufd1/Npl4 (Fig. 6*A*): unUb groove, UBS1 that is further divided into two sub-states, UBS2, and UBS3. The unUb groove accommodates an unfolded ubiquitin molecule along an Npl4 surface composed of conserved residues as previously described (28). UBS1 represents sites where ubiquitin is observed in two mutually exclusive positions in two distinctly folded states (Fig. 6*B*) while UBS2 and UBS3 each contribute a helix along with additional elements that interact with surfaces of tandem-folded ubiquitin molecules in the K48-linked chain (Fig. 6*C*). Interactions between SUMO and Ufd1 could not be resolved in EM maps, perhaps because the SIM is connected to Ufd1 through an extended and presumably disordered linker (last structured Ufd1 residue observed is amino acid 311; Ufd1 SIM is residues 357 to 361).

Cdc48 forms a hexameric base with its D1 and D2 AAA+ ATPase domains coming together as a double-stacked ring. Each ATPase domain is nucleotide-bound. In substrate-interacting and ubiquitin-unfolded classes, all but one D1 domain is bound to ATP, while all D2 domains were bound to adenosine diphosphate (ADP). In the substrate-unbound class, all D1 domains are bound to ATP and all D2 domains are bound to ADP. Most N-domains of Cdc48 were only visible at a lower threshold, although sufficient densities enabled atomic models to be traced for one or two intact N-domains along with portions of the others. While additional details and interactions with substrate are revealed, it is important to note that the overall configuration of complexes with respect to the D1 ring, Ufd1/Npl4 tower, and unfolded ubiquitin observed in our structures is consistent with previous structures of Ufd1/Npl4 with di-ubiquitin and the Ufd1/Npl4/Cdc48 complex with a polyubiquitin-modified substrate (28, 37) (Fig. 5*A*). With that said, the nucleotide occupancy and conformation of D2 rings in our structures with unfolded ubiquitin differ perhaps because we used WT Cdc48 while others used Cdc48 with a Walker B mutation to slow translocation (28). Comparison shows that our structures with unfolded ubiquitin exhibit a symmetrical D2 ring with EM densities consistent with ADP in each protomer while a structure with mutated Cdc48 (28, RCSB 6oa9) reveals different nucleotide-bound states, asymmetry in the ring, and interactions with unfolded ubiquitin via loops emerging from some of the respective Cdc48 protomers, contacts that are not observed in our analogous structures (*SI Appendix,* Fig. S6). The symmetric D2 rings in our structures are likely the result of ATP hydrolysis during sample preparation and do not represent a substrate translocating state.

Along with Npl4 residues 106 to 581, Ufd1 residues 228 to 256 and 281 to 311 are now resolved in our structures and form a tower atop the central pore of the Cdc48 hexamer. Other major differences observed between resolved substrate-interacting and ubiquitin-unfolded classes are the orientation of the tower relative to Cdc48 and the positioning of Npl4 residues 437 to 451, which forms a loop atop the Cdc48 central pore where substrate enters the hexamer during unfolding. The sequence and structure of this loop is highly conserved within yeast and human Npl4, especially around the “tip” of the loop. In unbound and substrate-bound states, this loop is positioned over the central pore where it could clash with substrate as its being unfolded. In ubiquitin-unfolded states, this loop moves away from the central pore to a position that allows substrate access to the central pore (Fig. 5*C*). In ubiquitin-unfolded states, the Npl4 tower appears perpendicular to the hexamer plane, but in unbound and substrate-bound states, the Ufd1/Npl4 tower tilts back and away from the Cdc48 central pore (*SI Appendix*, Fig. S7*A*). Gross motions of the Npl4 tower were previously associated with unfolding activity of the complex (38); however, it appears more likely that the tower is responding to the movement and asymmetry among subunits of the Cdc48 hexamer located just below the Npl4/Ufd1 tower (*SI Appendix*, Fig. S7*B*), especially between the two Cdc48 protomers that directly contact the unfolded substrate as it passes into the pore (*SI Appendix*, Fig. S7 *B* and *C*).

Resolved interactions with a distal K48-linked ubiquitin bound to UBS3 (see Fig. 5*B*; models uD and intB) involve an Npl4 helix composed of two conserved glutamates followed by mostly aliphatic residues (Fig. 6*C*). The conserved glutamate Glu557 is positioned within hydrogen bonding distance of the backbone amide of Ala46 in ubiquitin, while Leu561 interacts with the hydrophobic patch of ubiquitin around Ile44 of the most distal ubiquitin of the K48-linked ubiquitin chain observed in our structures. In addition to interactions with the UBS3 helix, contacts also emanate from an Npl4 loop between residues 514 to 518 and include hydrophobic contacts between Phe517 and ubiquitin Ile44 as well as polar contacts between Asn518 and the ubiquitin backbone at amino acid 47. UBS2 helix interactions with ubiquitin were observed previously and include interactions between the hydrophobic patch around Ile44 of ubiquitin and UBS2 residues Met574 and Ile575, and polar interactions between ubiquitin residues Arg42 and Arg72 with Glu578 (28, 37). Additional interactions in UBS2 with ubiquitin, akin to UBS3, involve an Npl4 loop between residues 536 to 540 that contribute polar contacts between the backbone at residue 537 and ubiquitin Lys6 and hydrophobic contacts between Ile538 and ubiquitin His68. The UBS3 helix is similar in structure and sequence to UIMs (39–41) and together with UBS2 constitute tandem interaction motifs that are associated with preferences for polyubiquitin binding (42) (Fig. 6*C*). Structural differences were not observed between states that did or did not have density for ubiquitin at UBS3, so we posit that in addition to ubiquitin interactions with the Ufd1 UT3 domain, UBS2 and UBS3 interactions with tandem K48-linked ubiquitin molecules anchor the chain at positions distal to the substrate while proximal ubiquitin proteins are unfolded.

The position of ubiquitin in UBS1 emerges as the most varied in our structures (Fig. 6*B*). The β1–β2 loop of ubiquitin nestles into a conserved “pivot groove” formed by Npl4 residues 285-300 (Fig. 6*B*). With the β1–β2 loop coordinated at this site, ubiquitin occupies at least two states, UBS1-A (akin to “Ub^prox^” in the structure of Ufd1/Npl4 in association with K48-linked di-ubiquitin (37), here shown within the context of the full complex with Cdc48) or UBS1-B [akin to “Ub^1^” in a structure of the full complex in association with a polyubiquitin-modified substrate (28)] (Fig. 6*B*). Distances between distal ubiquitin bound to UBS2 and Lys48 of ubiquitin bound to UBS1-A and UBS1-B are consistent with a covalent bond between these K48-linked ubiquitin molecules, although densities for the linkage were only observed between ubiquitin molecules bound at UBS1-A and UBS2. While the atomic models built for substrate-interacting states only had ubiquitin in UBS1-A (intA, intB), analysis of the EM density for substrate-interacting state intC suggests that ubiquitin can occupy UBS1-B prior to ubiquitin unfolding (*SI Appendix*, Fig. S8). The scarcity of particles in this state may attest to the transient nature of this intermediate in the pathway.

Ubiquitin in UBS1-A interacts with the previously described “N-loop” of Npl4 consisting of hydrophobic interactions between ubiquitin residues Val70, Ile71, and Leu8 and Npl4 Ala494, Met495, and Gly496 and polar contacts between ubiquitin Arg42 and Npl4 Ser498 (Fig. 6*B*) (37). Unlike UBS1-A, interactions between ubiquitin and UBS1-B involve alternate contacts to Npl4 as well as contacts to amino acid side chains emanating from segments of Ufd1 that were not previously modeled in substrate-bound structures of Ufd1/Npl4/Cdc48 (Fig. 6*B*). The β1–β2 loop of ubiquitin establishes polar interactions between ubiquitin Thr9 and the backbone amide of Npl4 Asp299, and between ubiquitin Thr7 and the imidazole ring of Npl4 His290; these interactions are not present at UBS1-B, though Thr9 may form polar interactions with the imidazole ring of His290 (Fig. 6*B*). Ubiquitin at UBS1-B makes additional interactions with Ufd1/Npl4, including ubiquitin Arg72 and Npl4 Tyr254 and ubiquitin Gln40 and Npl4 Gln293 that appear within hydrogen bonding distance while ubiquitin Glu34, Npl4 Glu292, and Ufd1 Arg241 and Ser237 form clusters of polar interactions (Fig. 6*B*). Interactions among these residues are remodeled when ubiquitin moves from UBS1-A to UBS1-B (Fig. 6*B*).

**Fig. 7.**
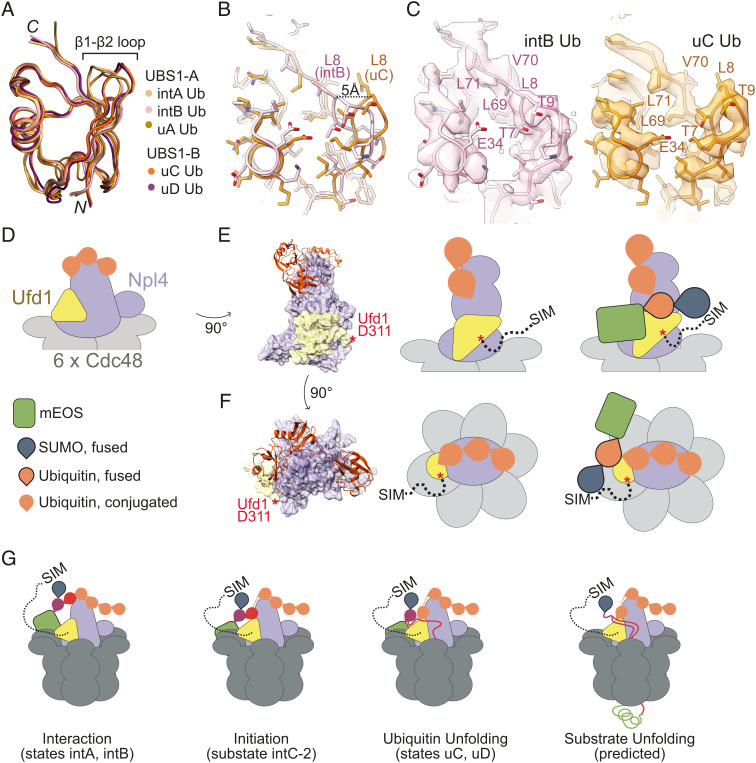
Unfolding by Ufd1/Npl4/Cdc48. (*A*) Ubiquitin in UBS1 in states A and B are superposed in ribbon representation color coded for each indicated structure. Amino and carboxy-terminal residues labeled N and C, respectively, with a bracket indicating locations of the β1–β2 loop. (*B*) Close-up of the model of ubiquitin at the C-terminal face of the ubiquitin fold with side chains in stick representation with a black dotted line indicating distance between α-carbons of Leu8 in intB (pink) and uC (orange). (*C*) EM densities and model with side chains in stick representation of ubiquitin from states intB (*Left*) and uC (*Right*). Select side chains are labeled. (*D*) Schematic of Ufd1/Npl4 and substrate in state intB. Throughout the figure, Ufd1 is represented in yellow, ubiquitin in orange, Npl4 in light blue, and Cdc48 in grey. Two views of the Ufd1/Npl4 in association with the substrate are shown—rotated 90° (*E*) and from above (*F*). A surface representation of Ufd1/Npl4 and a cartoon representation of ubiquitin (*Left*), a cartoon scheme of each view (*Middle*), and cartoon scheme with substrate (*Right*) are shown to illustrate positioning of the Ufd1/Npl4/Cdc48 complex relative to the substrate. Ufd1 residues 310 to 361 are indicated to approximate scale for an extended chain by a dotted black line with the “SIM” labeled at the C-terminal end. Red asterisk (*) denotes the last modeled residue of Ufd1. (*G*) Model for Ufd1/Npl4/Cdc48 unfolding a 1S1UH-mEOS substrate suggesting order to events left to right. Ubiquitin shown in different colors (purple, red, orange) to indicate proximity to the substrate.

Compared to ubiquitin bound to UBS1-A, interactions at USB1-B appear to anchor and separate the β1–β2 loop from the rest of ubiquitin as exemplified by a 5 Å displacement of Leu8 from the hydrophobic core (Fig. 7 *A* and *B*). These changes expose Leu69, Val70, and Leu71 of the ubiquitin hydrophobic core and result in a 150 Å^2^ increase of solvent-exposed surface area (Fig. 7*C*). Combined with the proximity of ubiquitin in UBS1-B relative to the unUb groove, and the presence of a sub-state with ubiquitin at UBS1-B prior to unfolding, these changes suggest that UBS1-B may contribute to destabilization of ubiquitin to initiate ATP-independent unfolding and capture of the ubiquitin N-terminal residues by Ufd1/Npl4/Cdc48. In previous time-resolved hydrogen deuterium exchange mass spectrometry experiments, peptides containing the β1–β2 loop incorporate deuterium faster than other peptides, regardless of nucleotide present (29), suggesting that destabilization of β1–β2 represents an early stage during ubiquitin unfolding. This hypothesis is further supported by studies suggesting that ubiquitin unfolding can be initiated by disrupting β1–β2 to dismantle the ubiquitin β-sheet (43–45).

## Discussion

The Ufd1/Npl4/Cdc48 complex is an established player in the ubiquitin–proteasome system that extracts polyubiquitylated proteins for recycling or to facilitate proteasomal degradation. Our findings support an additional layer of control that results in the prioritization of substrates for unfolding by the Ufd1/Npl4/Cdc48 complex with the SIM of Ufd1 conferring a preference for targets that are dually modified with SUMO and polyubiquitin over substrates containing only polyubiquitin.

Preferential unfolding of SUMO–polyubiquitin substrates in vitro may provide clues as to how Ufd1/Npl4/Cdc48 might prioritize substrates in the cell. Indeed, the observation that Ufd1/Npl4/Cdc48 favors hybrid SUMO–polyubiquitin substrates suggests that SUMO may not only serve as a signal to recruit Ufd1/Npl4/Cdc48 to sites of high SUMO densities, but that it could contribute to clearing SUMO–polyubiquitin-modified substrates prior to polyubiquitin-modified substrates after events such as heat shock or DNA damage. This may allow for rapid turnover of factors after early recruitment during these processes. It also is noteworthy that polyubiquitin substrates 1U^H^ and 2U^H^ are unfolded at different rates, an observation consistent with studies showing that different or more complex chain topologies impact processing by the proteasome (46). Thus, both chain topology and content serve as discriminating signals for unfolding by the Ufd1/Npl4/Cdc48 complex.

Structures of Ufd1/Npl4/Cdc48 in complex with the 1S1U^H^-mEOS substrate reveal states for ubiquitin bound to the complex prior to and during unfolding. Our structures with unfolded ubiquitin are consistent with prior work that showed how polyubiquitin-conjugated substrates associate with Ufd1/Npl4/Cdc48 during unfolding (28, 29), and reveal a previously uncharacterized UBS3 that appears to anchor distal ubiquitin in the ubiquitin chain. Our structures also show that ubiquitin can occupy different positions within UBS1 prior to and during ubiquitin unfolding, suggesting that binding of ubiquitin to UBS1-B and destabilization of its β1–β2 loop might precede ubiquitin unfolding (Fig. 7 *A–C* and *SI Appendix*, Fig. S8).

Ufd1/Npl4/Cdc48 can initiate unfolding of any ubiquitin within a K48-linked chain suggesting that a rate-limiting step may be the search for a substrate-proximal ubiquitin within the K48-linked chain before it can unfold the substrate (29). Our structures and the predicted topology of the 1S1U^H^-mEOS substrate relative to Ufd1/Npl4/Cdc48 and the Ufd1 SIM provide a plausible explanation for enhanced unfolding of SUMO–polyubiquitin substrates because interactions between SUMO and the Ufd1 SIM could position the substrate to restrict the search for substrate-proximal ubiquitin molecules within the K48-linked polyubiquitin chain (Fig. 7 *D*–*G*).

Ubiquitin and SUMO pathways are involved in several processes including the DNA damage response (12, 47, 48). Their convergence is especially evident in signaling by STUbL E3 ligases that promote ubiquitylation of SUMO or SUMO-modified proteins. The existence of ubiquitin, SUMO, SUMO–ubiquitin hybrid chains, and dual-modified substrates suggests that readers may exist to discriminate between these signals. In addition to human RAP80 which was shown to bind to SUMO and ubiquitin of dually modified proteins (23), we show that yeast Ufd1/Npl4/Cdc48 complexes are readers of substrates modified by hybrid chains, consistent with increased SUMO foci intensity and SUMO-conjugated proteins in vivo in the absence of the Ufd1 SIM (32, 33). It is also worth noting the existence of SUMO–polyubiquitin-specific ubiquitin proteases such as USP7 (49) and USP11 (50). Together, proteases could act in concert with Ufd1/Npl4/Cdc48 complexes to restore equilibrium or to terminate signaling by recognizing and resolving signals from SUMO–polyubiquitin hybrid modifications. While a C-terminal SIM is not apparent in human Ufd1, the human Cdc48 co-factor Fas-associated factor 1 (FAF1) has been implicated in SUMO recognition (51, 52), thus recognition and metabolism of SUMO–polyubiquitin hybrid substrates appear conserved in eukaryotic evolution.

## Methods

### Expressions and Purification of Ubiquitin, Uba1, Ube2K, and Slx8-Rfp2.

Ubiquitin (53), Uba1 (54), and Ube2K (55) were expressed and purified as previously described.

Genes of Slx8 and Rfp2 were cloned into MCS2 and MCS1 of pRSF-Duet1 vectors, respectively, and the plasmid was transformed into *Escherichia coli* BL21-CodonPlus (DE3)-RIL cells (Agilent Technologies) for expression. Cells were grown in Luria broth (LB) (Teknova) at 37 °C until OD_600_ reached 0.6, when expression was induced by addition of isopropyl-β-D-thiogalactopyranoside (IPTG) to 0.5 mM and lowering the temperature to 30 °C. Cells were harvested by centrifugation 4 h after induction.

Cells were suspended in a lysis buffer (1 μg/L DNase I, 1 mg/L lysozyme, 20 mM HEPES pH 8.0, 20% sucrose, 20 mM imidazole, 0.1% IGEPAL CA-630, 0.1 tris (2-carboxyethyl)phosphine (TCEP), and 0.1 mM PMSF) at 1 ml per gram of cells (wet weight) with 150 mM NaCl. After sonication, the lysate was centrifuged at 18,000 g for 40 min. The resulting supernatant was applied to Ni-NTA agarose beads in a column (ThermoFisher) equilibrated with wash buffer (20 mM HEPES pH 8.0, 150 mM NaCl, 20 mM imidazole, and 0.1 mM TCEP). The beads were washed with 5 column volumes of wash buffer and protein was eluted with 3 column volumes of elution buffer (wash buffer with 250 mM imidazole). Eluates were dialyzed to 20 mM HEPES pH 8.0, 150 mM NaCl, and 0.1 mM TCEP. Eluate was applied onto anion exchange with a MonoQ 10/100 GL column (Cytiva) equilibrated with the same buffer and eluted with a gradient to 60% of 20 mM HEPES pH 8.0, 1 M NaCl, and 0.1 mM TCEP.

### Expression, Purification, and Reconstitution of Ufd1, Npl4, and Cdc48.

Codon-optimized *S. cerevisiae* or *S. pombe* Ufd1, Ufd1^Δ^^SIM^ (Ufd1^1–256^), Npl4, and Cdc48 genes were inserted in the pTrx28 vector (56) and transformed into *E. coli* BL21-CodonPlus (DE3)-RIL cells for expression. Cells were grown at 37°C in Super Broth medium (Teknova) until OD_600_ reached 1.0. Expression was induced overnight at 18°C by addition of IPTG to 0.3 mM. Cells were harvested by centrifugation.

All purification steps for Ufd1, Npl4, and Cdc48 were done at 4 °C. Cell pellets were suspended in a lysis buffer (1 μg/L DNase I, 1 mg/L lysozyme, 20 mM Tris pH 8.0, 20% sucrose, 20 mM imidazole, 0.1% IGEPAL CA-630, and 0.1 mM TCEP) with 150 mM NaCl at a volume twice the pellet weight. Formation of Ufd1/Npl4 dimers or Ufd1/Npl4/Cdc48 complexes, lysates were mixed at this step at a 1:1:12 Ufd1:Npl4:Cdc48 ratio by volume. After sonication, lysate was centrifuged at 18,000 g for 40 min. The resulting supernatant was applied to a column of Ni-NTA agarose beads (ThermoFisher) equilibrated with wash buffer. The beads were washed with 5 column volumes of wash buffer and proteins eluted with 3 column volumes of elution buffer. Eluates were dialyzed to 20 mM HEPES pH 8.0, 150 mM NaCl, and 0.1 mM TCEP in the presence of tobacco etch virus (TEV) protease. If eluate contained Cdc48, 1 mM ATP and 5 mM MgCl_2_ were added at this step.

Proteins were separated using a HiLoad 26/60 Superdex 75 column (Cytiva) or HiLoad 26/60 Superdex 200 column (Cytiva) in the same buffer as dialysis.

### Expression and Purification of mEOS Variants.

The mEOS3.2 gene (Addgene) was cloned into the pET28-TEV vector using restriction enzyme cloning. Additional linear fusions of *S. cerevisiae* genes of Smt3-ubiquitin, 2(Smt3)-ubiquitin, 3(Smt3)-ubiquitin, and 2(ubiquitin) were cloned 5′ to generate additional N-terminal fusions with the Gibson Assembly Kit (New England Biolabs). Plasmids were transformed into *E. coli* BL-21 Codon Plus cells and grown in LB broth at 37°C until OD_600_ reached 0.6. Expression was induced overnight at 18°C with 0.3 mM IPTG. Cells were harvested by centrifugation.

The cell pellet was resuspended in lysis buffer with a final concentration of 350 mM NaCl. After sonication, the mixture was centrifuged at 18,000 g for 40 min. The resulting supernatant was applied to a column containing Ni-NTA beads equilibrated with wash buffer that included 350 mM NaCl. The beads were washed with 5 column volumes of wash buffer, and protein was eluted with 3 column volumes of elution buffer (wash buffer with 250 mM imidazole and 350 mM NaCl). Eluates were dialyzed to 20 mM HEPES pH 8.0, 150 mM NaCl, and 0.1 mM TCEP.

Proteins were further separated using an anion-exchange column (MonoQ column equilibrated with 20 mM HEPES pH 8.0, 150 mM NaCl, 0.1 mM TCEP eluted with a gradient to 60% of 20 mM HEPES pH 8.0, 1M NaCl, and 0.1 mM TCEP) followed by size-exclusion chromatography (Superdex 200 column equilibrated with 20 mM HEPES pH 8.0, 350 mM NaCl, and 0.1 mM TCEP).

### Biolayer Interferometry.

Ufd1/Npl4 (with WT Ufd1 or Ufd1^ΔSIM^) was biotinylated with 20-fold molar excess of Sulfo-NHS-Biotin (ThermoFisher Scientific) on ice for 1 h followed by size-exclusion chromatography to remove excess biotin. Measurements were performed with the Octet Red96e (Sartorius) biolayer interferometry instrument at standard kinetic settings (5.0 Hz) at 30 °C, shaking at 1,000 RPM.

Assays were conducted in Octet Kinetics Buffer (phosphate-buffered saline, 0.02% Tween-20, 0.1% bovine serum albumin (BSA), 0.1 mM TCEP) with biotinylated dimer immobilized on High Precision Streptavidin 2.0 Biosensors (Sartorius) and serial dilution of substrate (as indicated). Ligand and substrate concentrations were optimized for loading and signal range. These steps were followed: ligand (biotinylated dimer) loading until signal reached 2.0 nm, baseline step of 60 s, an association phase of 60 s, and a dissociation phase of 60 s. The data were corrected with alignment of datapoints to the average of the baseline step and to the dissociation step, with Savitzky–Golay filtering. Curves were fitted to a 2:1 heterogeneous ligand-binding model using the Data Analysis HT 12.0 program (Sartorius) to derive kinetic values. Multiple attempts were made to decrease nonspecific interactions with non-ubiquitin-conjugated substrate (as seen with the linear increase in signal during association) but were unsuccessful, so the best fit for a heterogeneous ligand was used to determine the kinetics.

### mEOS Substrate Ubiquitylation.

Conjugation reactions included 0.5 μM Uba1, 2 μM Ube2K, 2 μM Slx8-Rfp2, 5 μM of indicated base protein, and 50 μM ubiquitin and were incubated in 40 mM HEPES pH 8.0, 150 mM NaCl, 5 mM MgCl_2_, 0.1 mM TCEP, and 5 mM ATP for 2 h at 37 °C. The reactions were applied to a column of Ni-NTA beads, washed with 5 column volumes of wash buffer, and the polyubiquitylated substrate was eluted with 3 column volumes of elution buffer. Proteins were separated further by anion exchange (MonoQ) equilibrated at 20 mM HEPES pH 8.0, 100 mM NaCl, and 0.1 mM TCEP and eluted with a gradient to 60% with 20 mM HEPES pH 8.0, 1 M NaCl, and 0.1 mM TCEP. Fractions containing the protein of interest were pooled and fractionated by size-exclusion chromatography (S200 Increase) equilibrated at 20 mM HEPES pH 8.0, 150 mM NaCl, and 0.1 mM TCEP to separate proteins by polyubiquitin length.

### Substrate Photoconversion.

The mEOS substrates were photoconverted by irradiating at 365 nm on ice with the ENF-280C transilluminator (Spectroline) (57). For ubiquitylated substrates, photoconversion was carried out after ubiquitylation and purification. The substrates were irradiated until half of the proteins in molarity were photocleaved. Unless indicated otherwise, substrate concentrations refer to photocleaved concentrations as measured by absorbance at 571 nm.

### Single-Turnover Unfoldase Assay.

Assays under single-turnover employed an enzyme to substrate ratio of 10 to 1. 200 nM Ufd1/Npl4 or Ufd1^Δ^^SIM^/Npl4, 200 nM hexameric Cdc48, and 20 nM photocleaved substrate in unfoldase buffer (20 mM HEPES pH 8.0, 150 mM NaCl, 10 mM MgCl_2_, 1 mM BSA, 30 mM creatine phosphate, 3 U/mL phosphokinase, and 3 U/mL pyrophosphatase) was incubated at 30 °C for 10 min alone or in the presence of 1 μM Ulp1, 200 nM, or 2 μM Srs2^1107–1174^ where indicated. Reactions were initiated by addition of 5 mM ATP. Fluorescence was monitored using a SoftMax Pro 5 (Molecular Devices) at an excitation wavelength of 540 nm and an emission wavelength of 570 nm every second for 30 min at 30 °C.

The “percentage folded remaining” and initial rates (K_fast_, sec^−1^) were calculated by normalizing the data to background fluorescence decay (unfoldase reaction in the absence of ATP) and fitting the data to a two-phase exponential decay model using PRISM (GraphPad). *P* values were calculated using PRISM (GraphPad) by unpaired two-tailed *t *test or one-way ANOVA with Tukey’s test depending on number of conditions to compare; *P < 0.05, **P < 0.01, ***P < 0.001, ns (not significant).

### Multiple-Turnover Unfoldase Assay.

Assays under multiple-turnover employed an enzyme to substrate ratio of 1 to 8. 100 nM Ufd1/Npl4 or Ufd1^ΔSIM^/Npl4, 100 nM hexameric Cdc48, and 400 nM of each indicated substrate (for a net total of 800 nM substrate, photocleaved and non-cleaved, per reaction) were used. 1U^H^ was generated by incubating 1S1U^H^ with polyhistidine-tagged Ulp1, the latter of which was removed using nickel affinity chromatography. Buffer and assay conditions and analysis of results were the same as those for the single-turnover conditions, with the exception that “initial rate” was calculated from the first 30 s of unfolding, rather than K_fast_.

### Cryo-Electron Microscopy Sample Preparation and Data Collection.

A mix of 2 μM Ufd1/Npl4, 2 μM Cdc48, and 2.5 μM polyubiquitylated 1S1U^H^-mEOS was incubated on ice with 1 mM ATP in 20 mM HEPES pH 8.0, 150 mM NaCl, 0.1 mM TCEP, and 5 mM MgCl_2_ (*SI Appendix*, Fig. S3). A final concentration of 0.05% CHAPSO was added before vitrification. 4 μL of the sample was applied to a glow discharged UltrAuFoil R1.2/1.3 300 grid (Quantifoil), blotted for 4 s, and plunge frozen in liquid ethane using the Field Electron and Ion Company (FEI) Vitrobot Mark IV (ThermoFisher). This process was repeated three times using the same protein preparations but independently mixed and applied to a separate grid for each dataset. Data collection was carried out using a Titan Krios 300 kV (FEI) instrument equipped with a K3 Summit direct detector (Gatan). 10,793, 12,919, and 6,836 movies (40 frames/movie, 4 s exposure time) were collected in three sessions in super-resolution mode with a defocus range from −1.0 to −2.5 μm at a dose rate of ~ 20 e^−^/px/sec and a total dose of 72 e^−^/Å^2^/movie. The calibrated pixel size was 1.064 Å/px.

### Image Processing.

A summary of image processing steps is shown in *SI Appendix*, Fig. S4. Movies from each dataset were corrected for drift and dose-weighted with MotionCor2 in RELION 3.0 (58). Estimation of the contrast transfer function (CTF) was performed using Gctf (59). Movies with an estimated resolution worse than 4.0 Å, crystalline ice, or bad CTF fit were discarded. 500 particles were manually picked from a random subset of micrographs to obtain 2D classes that were used as templates for automated picking. Autopicked particles were extracted into 384 pixel boxes. Subsequent steps were carried out in cryoSPARC (60) for each dataset. Several rounds of 2D classification were done to remove junk particles. A 3D classification with image alignment using an ab-initio reconstruction with indicated class numbers (4, 2, and 3, respectively) was then done to remove particles that only contained Cdc48. Classes containing particles with Ufd1/Npl4/Cdc48 were selected and were combined for 3D refinement. Particle stacks from each dataset were used for 3D refinement and subsequent rounds of Bayesian polishing in RELION 3.0, after which the particles from the three datasets were combined for the next steps of image processing.

Using cryoSPARC, additional rounds of 2D classification and 3D classification were used to remove junk particles and to generate an initial reference model by ab initio reconstruction. 3D classification with four classes revealed three distinct classes; two with no substrate-bound (“substrate-unbound”), one with densities corresponding to unfolded ubiquitin (“ubiquitin-unfolded”) and one with densities for ubiquitin prior to unfolding (“substrate-interacting”). In the latter two classes, 3D variability analysis with 20 clusters or 3D classification was used to remove particles that did not have ubiquitin-bound or unfolded. For each substrate-containing class, a 3D refinement and focused refinement with a mask around the ATPase domains of Cdc48 were done to align particles and then density corresponding to Cdc48 were subtracted. The resulting particles were then subjected to 3D classification (with indicated number of classes in *SI Appendix*, Fig. S4) without image alignment to reveal different positions for ubiquitin.

The resulting classes (one substrate-unbound, three substrate-interacting, and four ubiquitin-unfolded) were subjected to 3D refinement and focused refinements with masks around density of the Cdc48 hexamer (entire hexamer and ATPase domains) and masks around density corresponding to the Ufd1/Npl4/substrate (central Ufd1/Npl4 and substrate, and upper portions of the density corresponding to the C-terminal region of Npl4 and folded ubiquitin densities). Resolutions reported in the text *SI Appendix*, Table S2 were calculated by RELION using the respective half maps and appropriate masks.

### Model Building and Refinement.

Atomic models were docked into maps and manually rebuilt in Coot (61). Prior cryo-EM structure of Cdc48 in complex with Npl4 (28) and ubiquitin (62) was used as starting models for Cdc48, Npl4, and folded ubiquitin moieties while regions of Ufd1 were built based upon secondary structure predictions and fit into densities. Composite maps were generated in Python-based Hierarchical ENvironment for Integrated Xtallography (PHENIX) (63) with focused refinement maps and atomic models were refined using PHENIX (63) and model geometry analyzed using Molprobity (64). Local resolution maps were generated using PHENIX. Structures and maps shown in figures were rendered using ChimeraX (65) and PyMOL (Schrödinger, LLC).

## Supplementary Material

Appendix 01 (PDF)

## Data Availability

EM data and refined coordinates are deposited and available in the Protein Data Bank (https://www.rcsb.org/) and Electron Microscopy Data Bank (https://www.ebi.ac.uk/emdb/) under accession codes 8DAR (substrate-unbound), 8DAS (intA), 8DAT (intB), 8DAU (uA), 8DAV (uC), and 8DAW (uD).

## References

[1] L. Cappadocia, C. D. Lima, Ubiquitin-like protein conjugation: Structures, chemistry, and mechanism. Chem. Rev. **118**, 889–918 (2018), 10.1021/acs.chemrev.6b00737. 28234446 PMC5815371

[2] J. R. Gareau, C. D. Lima, The SUMO pathway: Emerging mechanisms that shape specificity, conjugation and recognition. Nat. Rev. Mol. Cell Boil. **11**, 861–871 (2010), 10.1038/nrm3011. PMC3079294 21102611

[3] A. Flotho, F. Melchior, Sumoylation: A regulatory protein modification in health and disease. Annu. Rev. Biochem. **82**, 357–385 (2013), 10.1146/annurev-biochem-061909-093311. 23746258

[4] D. Komander, M. Rape, The ubiquitin code. Annu. Rev. Biochem. **81**, 203–229 (2012), 10.1146/annurev-biochem-060310-170328. 22524316

[5] J. R. Danielsen , DNA damage–inducible SUMOylation of HERC2 promotes RNF8 binding via a novel SUMO-binding Zinc finger. J. Cell Biol. **197**, 179–187 (2012), 10.1083/jcb.201106152. 22508508 PMC3328386

[6] N. Ellis , RNF4 regulates the BLM helicase in recovery from replication fork collapse. Front. Genet. **12**, 753535 (2021), 10.3389/fgene.2021.753535. 34868226 PMC8633118

[7] J. Keiten-Schmitz, K. Schunck, S. Müller, SUMO chains rule on chromatin occupancy. Front. Cell Dev. Biol. **7**, 343 (2020), 10.3389/fcell.2019.00343. 31998715 PMC6965010

[8] N. Martin , PARP-1 transcriptional activity is regulated by sumoylation upon heat shock. EMBO J. **28**, 3534–3548 (2009), 10.1038/emboj.2009.279. 19779455 PMC2782092

[9] M. H. Tatham , RNF4 is a poly-SUMO-specific E3 ubiquitin ligase required for arsenic-induced PML degradation. Nat. Cell Biol. **10**, 538–546 (2008), 10.1038/ncb1716. 18408734

[10] A. C. O. Vertegaal, Signalling mechanisms and cellular functions of SUMO. Nat. Rev. Mol. Cell Biol. **23**, 715–731 (2022), 10.1038/s41580-022-00500-y. 35750927

[11] Y. Yin , SUMO-targeted ubiquitin E3 ligase RNF4 is required for the response of human cells to DNA damage. Genes Dev. **26**, 1196–1208 (2012), 10.1101/gad.189274.112. 22661230 PMC3371408

[12] A. M. Sriramachandran, R. J. Dohmen, SUMO-targeted ubiquitin ligases. Biochim. Biophys. Acta **1843**, 75–85 (2014), 10.1016/j.bbamcr.2013.08.022. 24018209

[13] H. Sun, J. D. Leverson, T. Hunter, Conserved function of RNF4 family proteins in eukaryotes: Targeting a ubiquitin ligase to SUMOylated proteins. EMBO J. **26**, 4102–4112 (2007), 10.1038/sj.emboj.7601839. 17762864 PMC2230674

[14] C. M. Hickey, M. Hochstrasser, STUbL-mediated degradation of the transcription factor MATα2 requires degradation elements that coincide with corepressor binding sites. Mol. Biol. Cell **26**, 3401–3412 (2015), 10.1091/mbc.E15-06-0436. 26246605 PMC4591686

[15] Y. Erker , Arkadia, a novel SUMO-targeted Ubiquitin ligase involved in PML degradation. Mol. Cell Biol. **33**, 2163–2177 (2013), 10.1128/MCB.01019-12. 23530056 PMC3648077

[16] D. Jalal, J. Chalissery, A. H. Hassan, Genome maintenance in Saccharomyces cerevisiae: The role of SUMO and SUMO-targeted ubiquitin ligases. Nucleic Acids Res. **45**, 2242–2261 (2017), 10.1093/nar/gkw1369. 28115630 PMC5389695

[17] J. Prudden , SUMO-targeted ubiquitin ligases in genome stability. EMBO J. **26**, 4089–4101 (2007), 10.1038/sj.emboj.7601838. 17762865 PMC2230673

[18] Z. Sha, T. Blyszcz, R. González-Prieto, A. C. O. Vertegaal, A. L. Goldberg, Inhibiting ubiquitination causes an accumulation of SUMOylated newly synthesized nuclear proteins at PML bodies. J. Biol. Chem. **294**, 15218–15234 (2019), 10.1074/jbc.RA119.009147. 31285264 PMC6802522

[19] Z. Wang, G. Prelich, Quality control of a transcriptional regulator by SUMO-targeted degradation. Mol. Cell Biol. **29**, 1694–1706 (2009), 10.1128/MCB.01470-08. 19139279 PMC2655623

[20] J. W. Westerbeck , A SUMO-targeted ubiquitin ligase is involved in the degradation of the nuclear pool of the SUMO E3 ligase Siz1. Mol. Biol. Cell **25**, 1–16 (2014), 10.1091/mbc.E13-05-0291. 24196836 PMC3873881

[21] R. J. Lumpkin , Site-specific identification and quantitation of endogenous SUMO modifications under native conditions. Nat. Commun. **8**, 1171 (2017), 10.1038/s41467-017-01271-3. 29079793 PMC5660086

[22] J. Schimmel , The ubiquitin-proteasome system is a key component of the SUMO-2/3 cycle. Mol. Cell Proteomics **7**, 2107–2122 (2008), 10.1074/mcp.M800025-MCP200. 18565875

[23] C. M. Guzzo, RNF4-dependent hybrid SUMO-ubiquitin chains are signals for RAP80 and thereby mediate the recruitment of BRCA1 to sites of DNA damage. Sci. Signal. **5**, ra88 (2012), 10.1126/scisignal.2003485. 23211528 PMC4131685

[24] M. Nie , Dual recruitment of Cdc48 (p97)-Ufd1-Npl4 ubiquitin-selective segregase by small ubiquitin-like modifier protein (SUMO) and Ubiquitin in SUMO-targeted ubiquitin ligase-mediated genome stability functions. J. Biol. Chem. **287**, 29610–29619 (2012), 10.1074/jbc.M112.379768. 22730331 PMC3436128

[25] S. Bergink , Role of Cdc48/p97 as a SUMO-targeted segregase curbing Rad51–Rad52 interaction. Nat. Cell Biol. **15**, 526–532 (2013), 10.1038/ncb2729. 23624404

[26] N. W. Bays, R. Y. Hampton, Cdc48-Ufd1-Npl4: Stuck in the middle with Ub. Curr. Biol. **12**, R366–R371 (2002), 10.1016/s0960-9822(02)00862-x. 12015140

[27] M. M. Olszewski, C. Williams, K. C. Dong, A. Martin, The Cdc48 unfoldase prepares well-folded protein substrates for degradation by the 26S proteasome. Commun. Biol. **2**, 29 (2019), 10.1038/s42003-019-0283-z. 30675527 PMC6340886

[28] E. C. Twomey , Substrate processing by the Cdc48 ATPase complex is initiated by ubiquitin unfolding. Science **365**, eaax1033 (2019), 10.1126/science.aax1033. 31249135 PMC6980381

[29] Z. Ji , Translocation of polyubiquitinated protein substrates by the hexameric Cdc48 ATPase. Mol. Cell **82**, 570–584.e8 (2021), 10.1016/j.molcel.2021.11.033. 34951965 PMC8818041

[30] A. A. Armstrong, F. Mohideen, C. D. Lima, Recognition of SUMO-modified PCNA requires tandem receptor motifs in Srs2. Nature **483**, 59–63 (2012), 10.1038/nature10883. 22382979 PMC3306252

[31] B. Pfander, G.-L. Moldovan, M. Sacher, C. Hoege, S. Jentsch, SUMO-modified PCNA recruits Srs2 to prevent recombination during S phase. Nature **436**, 428–433 (2005), 10.1038/nature03665. 15931174

[32] J. B. Køhler, M. L. M. Jørgensen, G. Beinoraité, M. Thorsen, G. Thon, Concerted action of the Ubiquitin-fusion degradation Protein 1 (Ufd1) and sumo-targeted ubiquitin ligases (STUbLs) in the DNA-damage response. PLoS One **8**, e80442 (2013), 10.1371/journal.pone.0080442. 24265825 PMC3827193

[33] J. B. Køhler , Targeting of SUMO substrates to a Cdc48-Ufd1-Npl4 segregase and STUbL pathway in fission yeast. Nat. Commun. **6**, 8827 (2015), 10.1038/ncomms9827. 26537787 PMC4667616

[34] J.-G. Lee , Crystal structure of the Ube2K/E2-25K and K48-linked di-ubiquitin complex provides structural insight into the mechanism of K48-specific ubiquitin chain synthesis. Biochem. Biophy. Res. Commun. **506**, 102–107 (2018), 10.1016/j.bbrc.2018.10.067. 30336976

[35] I. Cooney , Structure of the Cdc48 segregase in the act of unfolding an authentic substrate. Science **365**, 502–505 (2019), 10.1126/science.aax0486. 31249134 PMC7362759

[36] E. E. Blythe, K. C. Olson, V. Chau, R. J. Deshaies, Ubiquitin- and ATP-dependent unfoldase activity of P97/VCP•NPLOC4•UFD1L is enhanced by a mutation that causes multisystem proteinopathy. Proc. Natl. Acad. Sci. U.S.A. **114**, E4380–E4388 (2017), 10.1073/pnas.1706205114. 28512218 PMC5465906

[37] Y. Sato , Structural insights into ubiquitin recognition and Ufd1 interaction of Npl4. Nat. Commun. **10**, 5708 (2019), 10.1038/s41467-019-13697-y. 31836717 PMC6910952

[38] M. Pan , Seesaw conformations of Npl4 in the human p97 complex and the inhibitory mechanism of a disulfiram derivative. Nat. Commun. **12**, 121 (2021), 10.1038/s41467-020-20359-x. 33402676 PMC7785736

[39] K. Hofmann, L. Falquet, A ubiquitin-interacting motif conserved in components of the proteasomal and lysosomal protein degradation systems. Trends Biochem. Sci. **26**, 347–350 (2001), 10.1016/s0968-0004(01)01835-7. 11406394

[40] D. Scott, N. J. Oldham, J. Strachan, M. S. Searle, R. Layfield, Ubiquitin-binding domains: Mechanisms of ubiquitin recognition and use as tools to investigate ubiquitin-modified proteomes" in Proteomics, (Wiley-VCH Verlag, 2015), **vol. 15(5–6)**, pp. 844–861, 10.1002/pmic.201400341. 25327553

[41] P. Young, Q. Deveraux, R. E. Beal, C. M. Pickart, M. Rechsteiner, Characterization of two polyubiquitin binding sites in the 26 S protease subunit 5a* J. Biol. Chem. **273**, 5461–5467 (1998), http://www.jbc.org. 9488668 10.1074/jbc.273.10.5461

[42] J. J. Sims, R. E. Cohen, Linkage-specific avidity defines the lysine 63-linked polyubiquitin-binding preference of Rap80. Mol. Cell **33**, 775–783 (2009), 10.1016/j.molcel.2009.02.011. 19328070 PMC2709242

[43] M. Carrion-Vazquez , The mechanical stability of ubiquitin is linkage dependent. Nat. Struct. Biol. **10**, 738–743 (2003), 10.1038/nsb965. 12923571

[44] A. Irbäck, S. Mitternacht, S. Mohanty, Dissecting the mechanical unfolding of ubiquitin. Proc. Natl. Acad. Sci. U.S.A. **102**, 13427–13432 (2005), 10.1073/pnas.0501581102. 16174739 PMC1224613

[45] A. K. Sahoo, B. Bagchi, P. K. Maiti, Unfolding dynamics of ubiquitin from constant force MD simulation: Entropy-enthalpy interplay shapes the free-energy landscape. J. Phys. Chem. B **123**, 1228–1236 (2019), 10.1021/acs.jpcb.8b09318. 30665306

[46] A. J. Boughton, S. Krueger, D. Fushman, Branching via K11 and K48 bestows ubiquitin chains with a unique interdomain interface and enhanced affinity for proteasomal subunit Rpn1. Structure **28**, 29–43.e6 (2020), 10.1016/j.str.2019.10.008. 31677892 PMC6996796

[47] D. A. Pérez Berrocal, K. F. Witting, H. Ovaa, M. P. C. Mulder, Hybrid chains: A Collaboration of ubiquitin and ubiquitin-like modifiers introducing cross-functionality to the ubiquitin code. Front. Chem. **7**, 931 (2019), 10.3389/fchem.2019.00931. 32039151 PMC6987259

[48] V. Rodriguez, R. Bailey, M. Larion, M. R. Gilbert, Retinoid receptor turnover mediated by sumoylation, ubiquitination and the valosin-containing protein is disrupted in glioblastoma. Sci. Rep. **9**, 16250 (2019), 10.1038/s41598-019-52696-3. 31700049 PMC6838077

[49] E. Lecona , USP7 is a SUMO deubiquitinase essential for DNA replication. Nat. Struct. Mol. Biol. **23**, 270–277 (2016), 10.1038/nsmb.3185. 26950370 PMC4869841

[50] I. A. Hendriks, J. Schimmel, K. Eifler, J. V. Olsen, A. C. O. Vertegaal, Ubiquitin-specific protease 11 (USP11) deubiquitinates hybrid small ubiquitin-like modifier (SUMO)-ubiquitin chains to counteract RING finger protein 4 (RNF4). J. Biol. Chem. **290**, 15526–15537 (2015), 10.1074/jbc.M114.618132. 25969536 PMC4477612

[51] A. Franz , USP7 and VCPFAF1 define the SUMO/Ubiquitin landscape at the DNA replication fork. Cell Rep. **37**, 109819 (2021), 10.1016/j.celrep.2021.109819. 34644576 PMC8527565

[52] C. H. Wang , Identification of two independent SUMO-interacting motifs in Fas-associated factor 1 (FAF1): Implications for mineralocorticoid receptor (MR)-mediated transcriptional regulation. Biochim. Biophys. Acta Mol. Cell Res. **1866**, 1282–1297 (2019), 10.1016/j.bbamcr.2019.03.014. 30935967

[53] S. Raasi, C. M. Pickart, "Ubiquitin chain synthesis" in Ubiquitin-Proteasome Protocols (Humana Press, 2005), vol. **301**, pp. 47–56, 10.1385/1-59259-895-1:047. 15917625

[54] Z. S. Hann , Structural basis for adenylation and thioester bond formation in the ubiquitin E1. Proc. Natl. Acad. Sci. U.S.A. **116**, 15475–15484 (2019), 10.1073/pnas.1905488116. 31235585 PMC6681703

[55] C. M. Pickart, S. Raasi, "Controlled synthesis of polyubiquitin chains" in Methods in Enzymology, (Elsevier, 2005), **vol. 399**, pp. 21–36. 10.1016/S0076-6879(05)99002-2. 16338346

[56] L. Cappadocia, A. Pichler, C. D. Lima, Structural basis for catalytic activation by the human ZNF451 SUMO E3 ligase. Nat. Struct. Mol. Biol. **22**, 968–975 (2015), 10.1038/nsmb.3116. 26524494 PMC4709122

[57] S. A. McKinney, C. S. Murphy, K. L. Hazelwood, M. W. Davidson, L. L. Looger, A bright and photostable photoconvertible fluorescent protein. Nat. Methods **6**, 131–133 (2009), 10.1038/nmeth.1296. 19169260 PMC2745648

[58] J. Zivanov , New tools for automated high-resolution cryo-EM structure determination in RELION-3. Elife **7**, e42166 (2018), 10.7554/eLife.42166. 30412051 PMC6250425

[59] K. Zhang, Gctf: Real-time CTF determination and correction. J. Struct. Biol. **193**, 1–12 (2016), 10.1016/j.jsb.2015.11.003. 26592709 PMC4711343

[60] A. Punjani, J. L. Rubinstein, D. J. Fleet, M. A. Brubaker, cryoSPARC: Algorithms for rapid unsupervised cryo-EM structure determination. Nat. Methods **14**, 290–296 (2017), 10.1038/nmeth.4169. 28165473

[61] P. Emsley, B. Lohkamp, W. G. Scott, K. Cowtan, Features and development of Coot. Acta Crystallogr. D Biol. Crystallogr. **66**, 486–501 (2010), 10.1107/S0907444910007493. 20383002 PMC2852313

[62] S. Vijay-Kumar, C. E. Bugg, W. J. Cook, Structure of ubiquitin refined at 1.8 A resolution. J. Mol. Biol. **194**, 531–544 (1987), 10.1016/0022-2836(87)90679-6. 3041007

[63] P. D. Adams , PHENIX: A comprehensive Python-based system for macromolecular structure solution. Acta Crystallogr. D Biol. Crystallogr. **66**, 213–221 (2010), 10.1107/S0907444909052925. 20124702 PMC2815670

[64] V. B. Chen , MolProbity: All-atom structure validation for macromolecular crystallography. Acta Crystallogr. D Biol. Crystallogr. **66**, 12–21 (2010), 10.1107/S0907444909042073. 20057044 PMC2803126

[65] E. F. Pettersen , UCSF ChimeraX: Structure visualization for researchers, educators, and developers. Protein Sci. **30**, 70–82 (2021), 10.1002/pro.3943. 32881101 PMC7737788

