# Supplementary material for: SUMO enhances unfolding of SUMO–polyubiquitin-modified substrates by the Ufd1/Npl4/Cdc48 complex
Source: Proc Natl Acad Sci U S A. 2022 Dec 27;120(1):e2213703120. doi: 10.1073/pnas.2213703120 (PMC9910466; doi:10.1073/pnas.2213703120)
Supplement: Supplementary file 1 — Appendix 01 (PDF) [file pnas.2213703120.sapp.pdf]

PNAS

Supporting Information for

SUMO enhances unfolding of SUMO-polyubiquitin-modified substrates by the Ufd1/Npl4/Cdc48 complex

Hyein G. Lee, Abigail A. Lemmon, and Christopher D. Lima*

Corresponding Author: Christopher D. Lima
Email: limac@mskcc.org

This PDF file includes:

Figures S1 to S8
Tables S1 to S2

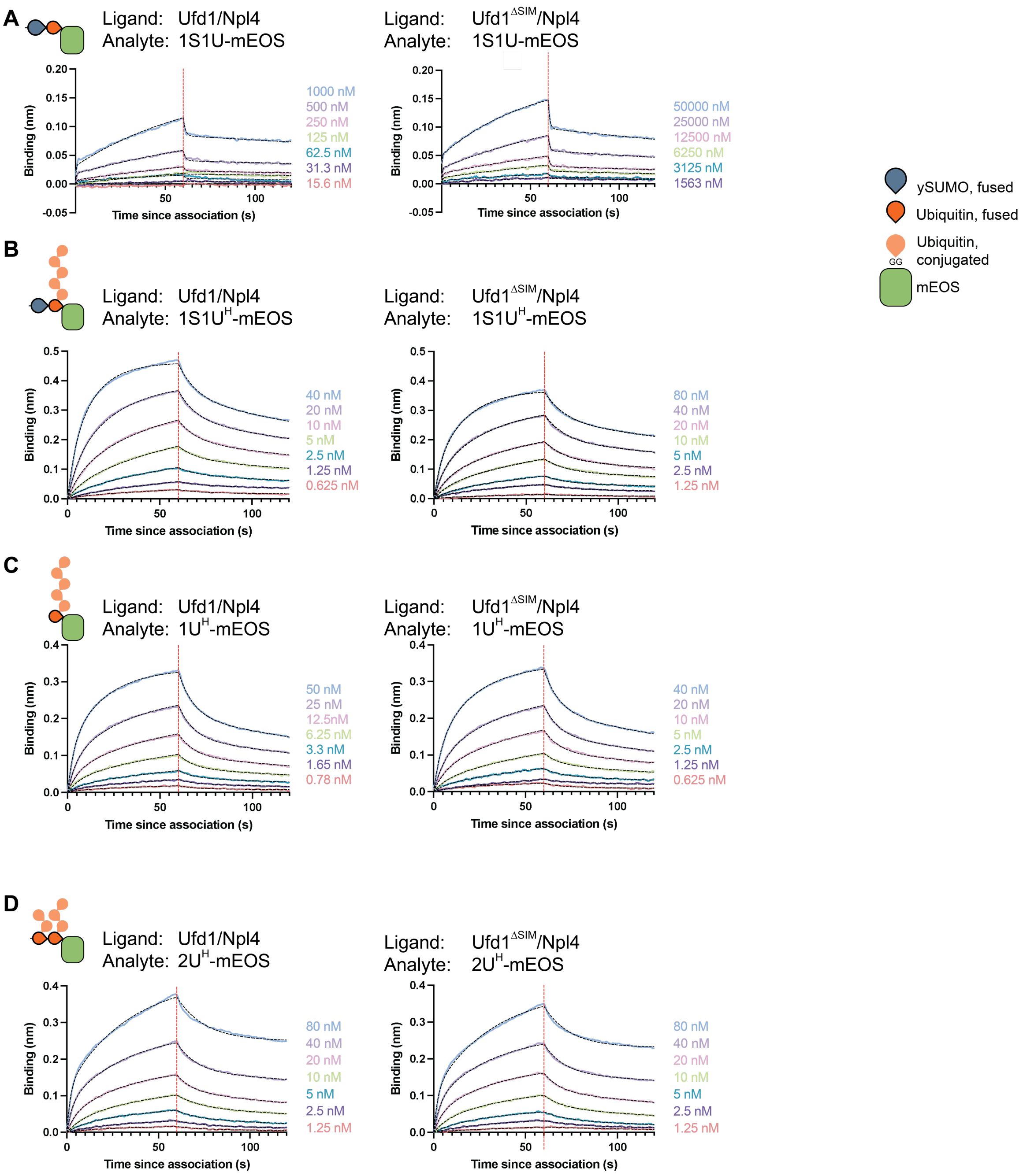

Fig. S1. Biolayer interferometry measurements. (A) Binding of wild-type and ΔSIM Ufd1/Npl4 to 1S1U-mEOS. (B) Binding of wild-type and ΔSIM Ufd1/Npl4 to 1S1U^H-mEOS. (C) Binding of wild-type and ΔSIM Ufd1/Npl4 to 1U^H-mEOS. (D) Binding of wild-type and ΔSIM Ufd1/Npl4 to 2U^H-mEOS. Time-dependent binding response curves shown with concentrations as in legend and global fit for 2:1 heterogeneous ligand binding model generated by Octet HT Data Analysis program shown as a dashed black curve. Dotted vertical red lines indicate end of association and beginning of dissociation phases.

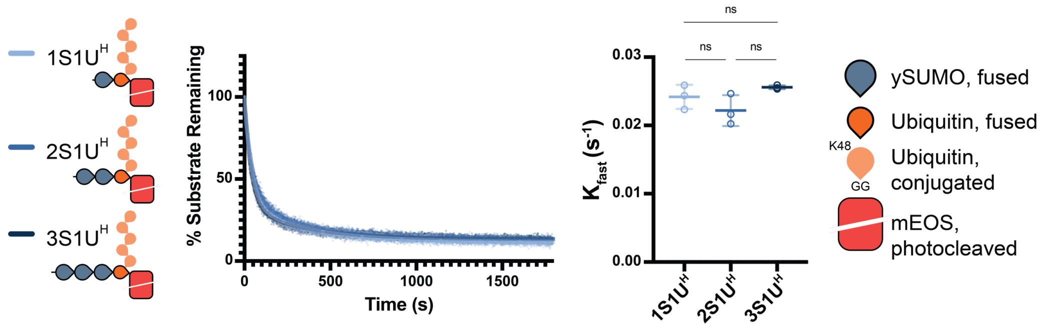

Fig. S2. Unfolding assay for $1S1U^H$, $2S1U^H$, and $3S1U^H$ by wild-type Ufd1/Npl4/Cdc48. Values were normalized to background fluorescence in the absence of ATP. Plot of three replicates with fit of two-phase non-linear regression. K_{fast} (sec^{-1}) determined using two phase decay fit. Error bars represent standard deviation. P values calculated by one-way ANOVA with Tukey's test; * ($P<0.05$), ** ($P<0.01$), *** ($P<0.001$), ns (not significant).

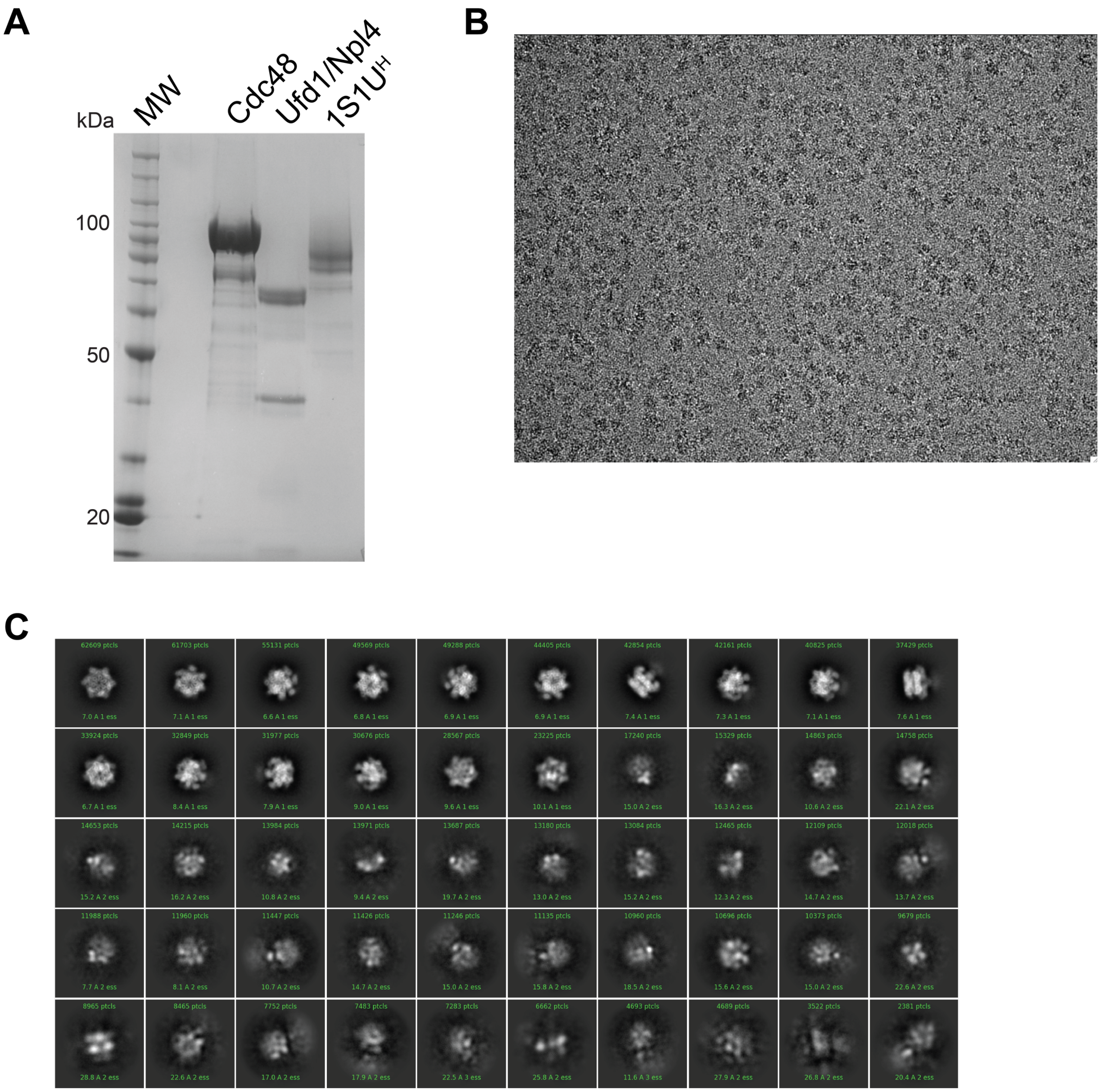

Fig. S3. Cryo-EM sample preparation and data collection. (A) SDS-PAGE analysis of components used in sample preparation. Molecular weight marker (MW) is Invitrogen BenchMark Protein Ladder with 20, 50 and 100 kDa shown on the left. (B) Example micrograph from data collection (dataset 1). (C) Output of initial 2D classification of particles.

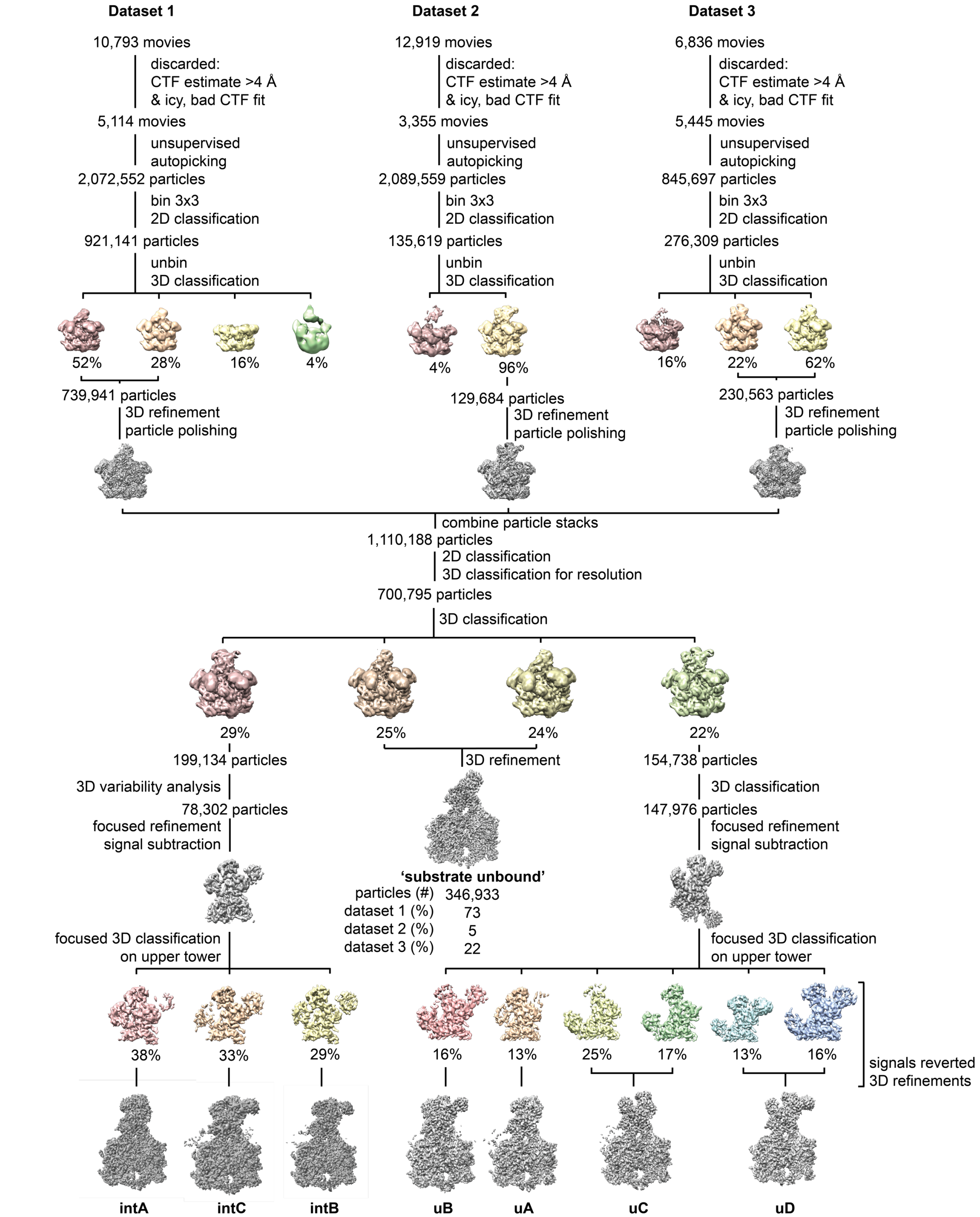

	intA	intC	intB	uB	uA	uC	uD
particles (#)	29,595	26,197	22,510	23,353	36,688	48,941	38,994
dataset 1 (%)	66	66	66	48	48	48	47
dataset 2 (%)	7	8	7	28	27	28	30
dataset 3 (%)	27	26	27	24	25	24	23
	'substrate interacting'			'ubiquitin-unfolded'			

Fig. S4. Cryo-EM analysis of Ufd1/Npl4/Cdc48 in presence of 1S1U^{H}-mEOS and ATP. Data collection, image processing, refinement, and classification steps shown along with images of reconstructions at each refinement step. Final particle count shown below each state along with the percentage of particles from each dataset for that state.

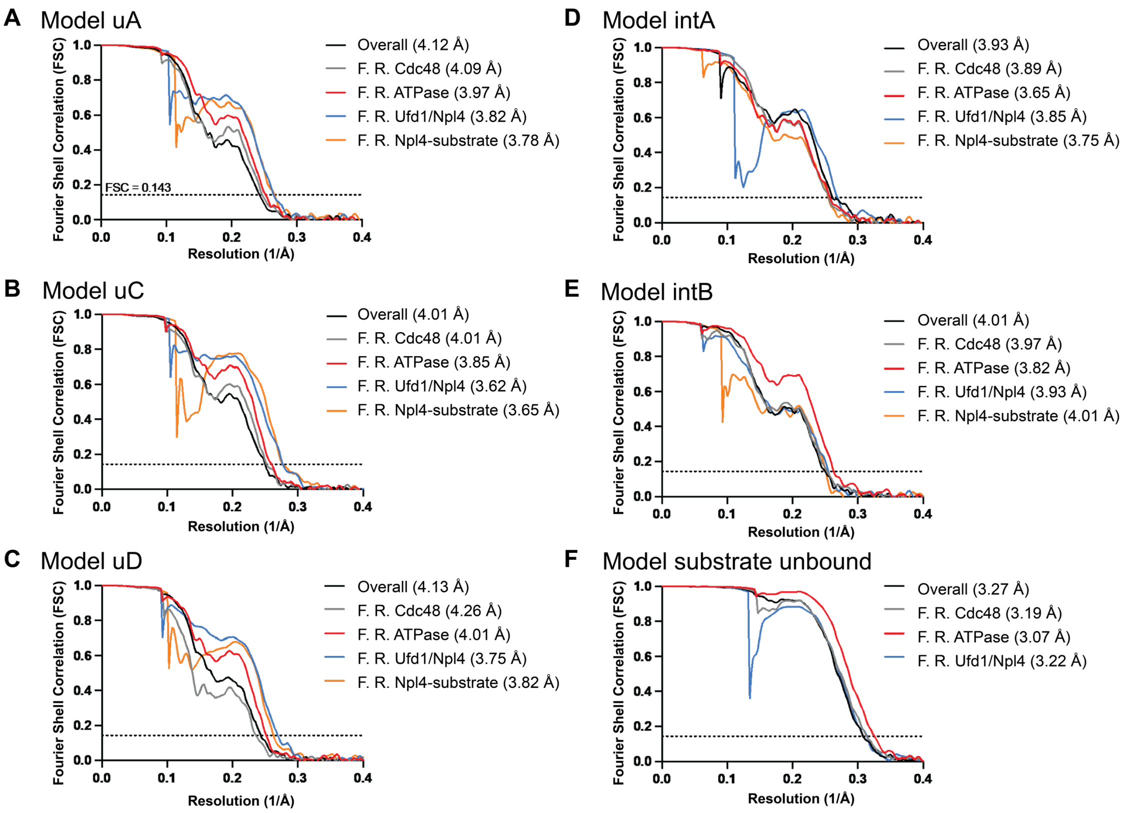

Fig. S5. FSC curves for cryo-EM reconstructions. Fourier Shell Correlation between half maps obtained from indicated reconstructions for ubiquitin unfolded state uA (A), uC (B), uD (C), substrate interacting state intA (D), intB (E), and substrate unbound state (F).

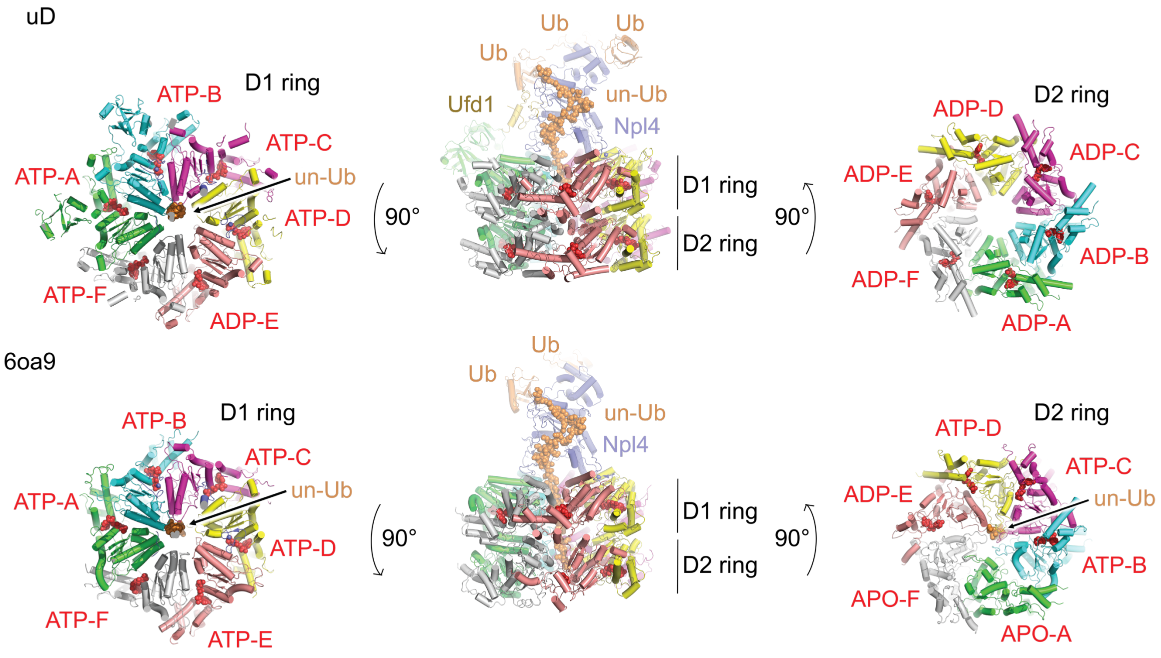

Fig. S6. Cdc48 comparison. Cartoon representation and comparison between Cdc48 rings using the ubiquitin unfolded state uD (top) and PDB 6oa9 (bottom), specifically comparing D1 rings and nucleotide occupancy (left), complex composition (middle), and D2 rings (right). Nucleotide identity and occupancy are indicated in red in left and right panels). Cdc48 is colored by protomer with nucleotide (red) and unfolded ubiquitin (light brown; un-Ub) shown as spheres.

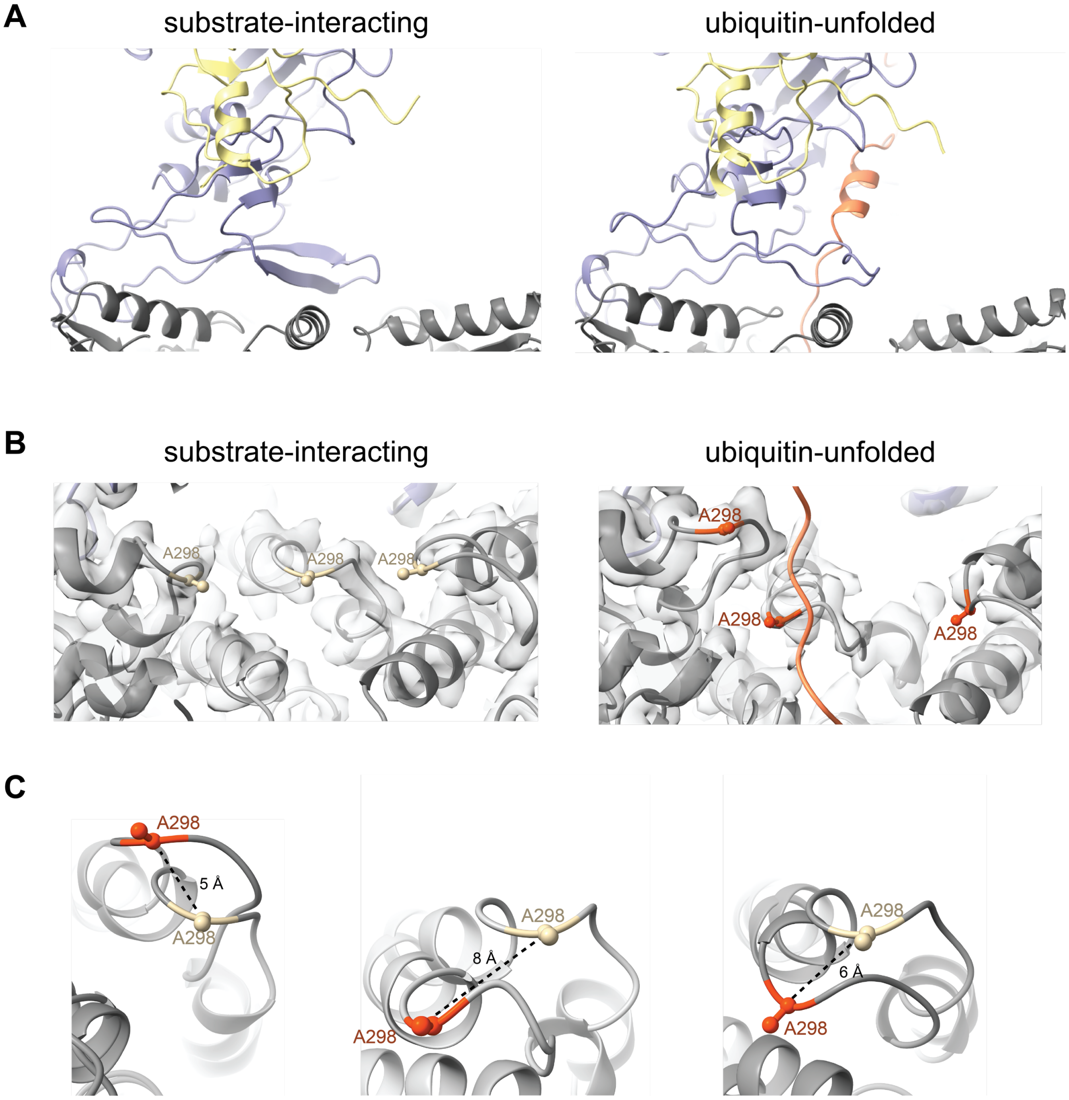

Fig. S7. Orientation of Cdc48 subunits in substrate-interacting and ubiquitin-unfolded classes. (A) Placement of Ufd1/Npl4 relative to the Cdc48 differs between substrate-interacting and ubiquitin-unfolded classes. Models shown are state intB (for substrate-interacting) and uC (for ubiquitin-unfolded). Npl4 is in light blue, Ufd1 is in yellow, Cdc48 is in grey, and unfolded ubiquitin is in orange. (B) Relative positioning of Cdc48 monomers with the position of a loop containing Ala298 indicated in the region of Cdc48 that contacts substrate as its being unfolded. In substrate unbound or substrate-interacting classes the Cdc48 hexamer is symmetric and the loops containing Ala298 (colored beige) of respective Cdc48 protomers are in the same plane. In ubiquitin-unfolded classes, adjacent Cdc48 protomers move relative to one another shifting the loop containing Ala298 (colored in red) out of this plane. (C) Close up of the loop containing Ala298 in substrate-interacting class (beige) and ubiquitin unfolded class (red) overlayed in three monomers of Cdc48 indicating the relative change in position of Ala298 within these Cdc48 loops.

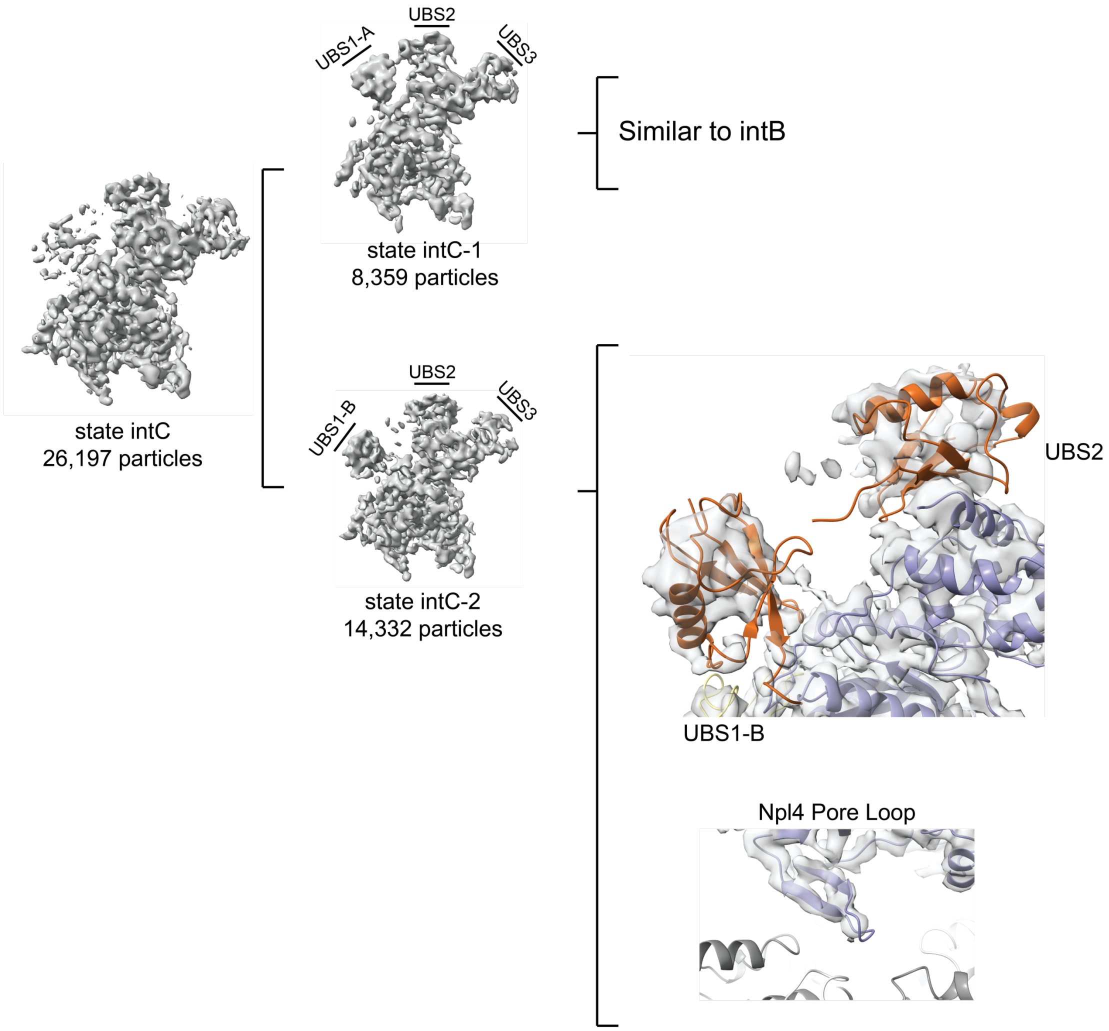

Fig. S8. Subclassification of state intC. 3D classification without image alignment of particles in state intC revealed two substates with densities for ubiquitin at UBS1-A or UBS1-B. Overlay of atomic models with the reconstructed map of intC-2 show ubiquitin molecules at positions UBS1-B and UBS2 and a closed conformation for the Npl4 loop, conformations not present in atomic models for intA and intB.

Table S1. Association and dissociation parameters of interaction between Ufd1/Npl4 and substrates containing SUMO and ubiquitin. K_D values derived from steady-state responses while K_a and K_{dis} values derived from association and dissociation kinetics fitted to a 2:1 kinetic binding model. Standard error shown.

**Ligand**	Ufd1$^{\Delta SIM}$/ Npl4	Ufd1/ Npl4	Ufd1$^{\Delta SIM}$/ Npl4	Ufd1/ Npl4	Ufd1$^{\Delta SIM}$/ Npl4	Ufd1/ Npl4	Ufd1$^{\Delta SIM}$/ Npl4	Ufd1/ Npl4
**Analyte**	2U^H-mEOS	2U^H-mEOS	1U^H-mEOS	1U^H-mEOS	1S1U^H-mEOS	1S1U^H-mEOS	1S1U-mEOS	1S1U-mEOS
**K_D 1 (nM)**	<0.001 ± 0.001	<0.001 ± <0.001	5.29 ± 0.06	5.71 ± 0.07	4.97 ± 0.10	2.42 ± 0.04	1.13*10^4 ± 300	193 ± 11
**K_D 2 (nM)**	15.7 ± 0.2	13.4 ± 0.2	40.7 ± 0.6	34 ± 0.4	34.2 ± 0.6	26.4 ± 0.5	1.38*10^4± 960	309 ± 39
**K_a 1 (x1,000/Ms)**	411 ± 4	384 ± 3	1020 ± 6	906 ± 5	704 ± 4	1760 ± 7	0.322 ± 0.008	11.8 ± 0.6
**K_a 2 (x1,000/Ms)**	4300 ± 46	4790 ± 51	2950 ± 40	3620 ± 40	2620 ± 36	3790 ± 61	81.7 ± 5	2770 ± 320
**K_{dis} 1(/s)**	1.54*10^{-7} ± 7*10^{-7}	<1.0*10^{-7} ± 3*10^{-7}	0.005 ± 6*10^{-5}	0.005 ± 6*10^{-5}	0.004 ± 7*10^{-5}	0.004 ± 6*10^{-5}	0.004 ± 4*10^{-5}	0.002 ± 7*10^{-5}
**K_{dis} 2(/s)**	0.068 ± 0.004	0.064 ± 0.0004	0.12 ± 0.0009	0.123 ± 0.0008	0.09 ± 0.001	0.1± 0.001	1.13 ± 0.03	0.857 ± 0.04

Table S2. Statistics for data collection and refinement.

**Data Collection**	Dataset 1		Dataset 2		Dataset 3	
Magnification	22,500x					
Voltage (kV)	300					
Electron exposure (e-/Å2)	72					
Defocus range (μm)	-1.0 to -2.5					
Super-resolution pixel size (Å)	0.532					
Fourier cropped pixel size (Å)	1.064					
Initial particle projections (#)	700,795					
Symmetry	C1					
	unbound	intA	intB	uA	uC	uD
PDB accession codes	8DAR	8DAS	8DAT	8DAU	8DAV	8DAW
Final particle projections (#)	346,933	29,595	22,510	36,688	49,941	38,994
Map res (Å) FSC threshold = 0.143						
Overall map (Å)	3.3	3.9	4.0	4.1	4.0	4.1
Focused Refinement: Cdc48 (Å)	3.8	3.9	4.0	4.1	4.0	4.3
ATPase (Å)	3.6	3.7	3.8	4.0	3.9	4.0
Ufd1/Npl4 tower (Å)		3.9	3.9	3.8	3.6	3.8
Npl4-substrate (Å)		3.8	4.0	3.8	3.7	3.8
Local resolution range						
Overall map (Å)	2.8 - 7.6	3.2 - 8.2	3.2 - 10.5	3.3 - 9.4	3.3 - 8.7	3.5 - 8.7
Focused Refinement: Cdc48 (Å)	2.8 - 6.6	3.3 - 10.0	3.3 - 10.0	3.4 - 8.4	3.4 - 7.7	3.5 - 8.3
ATPase (Å)	2.9 - 4.5	3.2 - 5.7	3.3 - 6.6	3.4 - 7.1	3.4 - 6.7	3.4 - 7.1
Ufd1/Npl4 tower (Å)	2.9 - 5.0	3.1 - 6.2	3.5 - 8.2	3.3 - 5.5	3.1 - 5.1	3.2 - 5.3
Npl4-substrate (Å)	N/A	3.1 - 4.9	3.6 - 6.0	3.2 - 5.0	3.0 - 4.8	3.3 - 5.4
**Refinement**						
Initial models used	6OA9, 1UBQ					
Model res (Å) FSC threshold = 0.5	3.3	3.9	4	4.1	3.8	3.9
Model composition						
Non-hydrogen atoms	30,157	34,059	34,635	34,248	34,248	34,848
Protein residues	3,827	4,315	4,388	4,342	4,342	4,418
Ligand (Zn)	2	2	2	2	2	2
Ligand (ATP)	6	5	5	5	5	5
Ligand (ADP)	6	7	7	7	7	7
Mean B factors						
Protein	55.57	123.03	134.51	149.12	121.15	143.49
Ligands	47.68	107.99	118.39	140.51	113.21	133.33
RMS deviations						
Bond lengths	0.004	0.003	0.003	0.003	0.004	0.004
Bond angles	0.603	0.553	0.534	0.932	0.958	0.985
Validation						
Molprobity score	1.88	1.96	1.84	1.87	1.85	1.87
Clash score	6.06	7.37	7.14	9.62	9.34	9.62
Rotamer Outliers (%)	1.93	1.89	1.34	0.90	0.79	0.48
C-beta deviations	0.00	0.00	0.00	0.25	0.25	0.22
Ramachandran plot						
% favored	95.33	95.07	95.01	94.67	94.81	94.64
% allowed	4.67	4.93	4.97	5.31	5.15	5.31
% outliers	0.00	0.00	0.00	0.02	0.05	0.05
